# Abstracts from the 8th International Conference for Healthcare and Medical Students (ICHAMS): Dublin, Ireland.

**DOI:** 10.1186/s12919-019-0167-8

**Published:** 2019-08-13

## A1: Attitudes of healthcare professionals working in primary healthcare centres towards interprofessional collaboration in Qatar

### Shimaa Aboelbaha, Alla El-Awaisi, Zeinab Abedini, Ahmed Awaisu

#### ^1^Department of Pharmacy, Qatar University, Doha, Qatar

##### **Correspondence:** Shimaa Aboelbaha

**Introduction:** Interprofessional collaboration (IPC) is defined as the active collaboration between health workers from various backgrounds to deliver the optimum quality of care to patients. Due to its important health impacts and lack of studies with similar scope in Qatar and the Middle East, this study has been conducted to explore the knowledge, awareness, experience, readiness and, attitude of healthcare practitioners (HCP) working in primary healthcare centres (PHCC) in Qatar towards IPC.

**Methods:** This study is a quantitative, cross-sectional, prospective, web-paper-based questionnaire survey. Surveys were sent to 2500 HCPs who understand English and are working in PHCCs in Qatar. Formulating questions were adopted from two different validated surveys used in previous research. The data were presented as means ± SD and analysed using Chi-square, and analysis of variance (ANOVA) tests.

**Results:** 1415 participants completed the survey with a response rate of 56.6 %. Participants indicated good awareness of the differences between interaction and collaboration, and that the highest collaboration is with physicians followed by nurses and pharmacists. Moreover, participants claimed that they have long experience with IPC. Results of the inferential analysis showed that females have a stronger attitude and readiness towards IPC than males, and physicians seemed to have more readiness towards IPC than other professionals (P<0.001).

**Discussion:** Results of this study showed positive attitudes towards IPC in the region. Main strength of the study is the multiple administration methods (web/paper surveys), and main limitation is the excessive survey length. Further studies are needed to explore attitudes of HCPs in advanced care facilities.

## A2: Audit determining effectiveness of discharge orders in a general medicine service

### Nikki Cliffe^1^, Seamus Sreenan^2^

#### ^1^School of Medicine, Royal College of Surgeons in Ireland, Dublin, Ireland, Dublin, Ireland; ^2^Connolly Hospital Blanchardstown, Dublin, Department of Endocrinology, Dublin, Ireland

##### **Correspondence:** Nikki Cliffe

**Introduction:** Outpatient follow-up succeeding hospital admission is a critical component of ongoing patient safety. Due to the effect of prolonged length of stay on healthcare economics and the incidence of adverse healthcare events, there is rising pressure on medical teams to discharge patients in a timely manner. This in turn leads to increased follow-up appointments and investigations to be completed in outpatient departments. It is thus of prime importance that the requested follow-up orders are scheduled upon, or shortly after, patient discharge.

**Methods:** Discharge orders from a general medicine service over a 12-month period at Connolly Hospital Blanchardstown were inspected for requested follow-up appointments and investigations. Subsequently, the patient administration, radiology and biolab systems were accessed to determine which appointments, radiology orders and laboratory tests, respectively, were actually scheduled.

**Results:** Interestingly, 21.0% of requested follow-up appointments and 21.0% of requested investigations were not scheduled. Of note, 28.7% of patients did not have all their requested follow-up appointments and investigations scheduled, highlighting a significant short-coming regarding patient follow-up after discharge.

**Discussion:** Ultimately, we have identified a quality gap in scheduling appointments and investigations following discharge of patients on the general medicine service. There are likely multiple factors contributing to this quality gap, which represents a risk to patient safety. A follow-up prompt sheet was developed as a quality improvement strategy and a re-audit in 12 months will determine its impact. It is hoped that the proposed solution will enhance the effectiveness of patient care and reduce this risk.

## A3: Anti-microbial peptides synthesis and the use of circular dichroism to observe their secondary structures

### Emaad Al Shibli^1^, Marc Devocelle^2^

#### ^1^Department of Chemistry, Royal College of Surgeons in Ireland (RCSI), Dublin 2, Ireland; ^2^RCSI, Department of Chemistry, Royal College of Surgeons in Ireland (RCSI), Dublin 2, Ireland

##### **Correspondence:** Emaad Al Shibli

**Introduction:** The emergence of antimicrobial resistance has led to the development of novel antimicrobial agents. Antimicrobial peptides (AMP) have been investigated as new antimicrobial agents. One of the limitations of the clinical use of AMPs is their narrow therapeutic indices. The use of AMPs as prodrugs could minimize unwanted effects of the active AMPs. In this study, we aimed to prove that one such AMP prodrug, Pro-WMR has less toxicity on human membranes than its active peptide counterpart by studying their secondary structures.

**Methods:** The AMP and its prodrugs were assembled with an automated microwave peptide synthesizer (Liberty Blue™). Then, Circular Dichroism (CD) was used to find out the presence or not of secondary structures in WMR and its prodrug, in the absence or presence of an organic solvent stabilizing these structures.

**Results:** Pro-WMR had a random coil when interacting with buffer and a slight propensity to form a helix when interacting with membrane mimicking environment. On the other hand, active WMR showed an active alpha helix and random coil in the presence and absence of an organic solvent respectively.

**Discussion:** The effects of the prodrug modification on WMR, indicate that the loss of activity originates from charge reduction rather than inhibition of secondary structure formation. The modification of AMPs as prodrugs could reduce their toxicity and address the clinical limitations associated with their therapeutic indices. The present work provides further information on AMPs conformational interactions with both human and microbial membranes and contributes to elucidate their mechanism of action.

## A4: Correlation of matrix metalloprotease activity with clinical outcome in cystic fibrosis via the CF-ABLE score

### Isha Bagwe^1^, Oliver McElvaney^2^, Emer Reeves^2^, Noel McElvaney^2^

#### ^1^Respiratory Medicine, The Royal College of Surgeons, Dublin, Ireland; ^2^Department of Medicine, The Royal College of Surgeons, Dublin, Ireland

##### **Correspondence:** Isha Bagwe

**Introduction:** Cystic fibrosis (CF) results from mutations in the cystic fibrosis trans-membrane conductance regulator (CFTR) gene and is characterised by neutrophilic airway inflammation. Matrix metalloproteases (MMP) require activation by extracellular proteases (e.g. neutrophil elastase (NE)) in order to degrade extracellular matrix proteins. The CF-ABLE score predicts the risk of death or requirement for lung transplantation, using four clinical parameters; age, body mass index, forced expiratory volume in 1 second (FEV1) and frequency of exacerbations. This study aimed to further validate the CF-ABLE score by correlating CF-ABLE score and MMP activity.

**Methods:** Beaumont Hospital Ethics Committee granted ethical approval. Levels of pro-MMP and cleaved MMP in bronchoalveolar lavage (BAL) and sputum from people with CF (PWCF, n=30), healthy controls (HC, n=3) and F508del/F508del PWCF post double-lung transplant (DLT, n=3) were assessed by Western Blot. MMP and NE activity were measured by fluorescence resonance energy transfer assay.

**Results:** MPP9 levels correlated significantly with CF-ABLE score (P=0.04). MMP activity correlated strongly with CF-ABLE score (R2=0.81,P<0.0001), FEV1 (R2=0.69,P=0.01) and NE activity (R2=0.84,P<0.0001). CFTR potentiator, Ivacaftor, did not affect cleaved MMP levels in PWCF, who had higher cleaved MMP levels than HC (P=0.01), indicating that this effect is independent of CFTR dysfunction. Post-DLP, cleaved MMP levels normalized, implicating chronic pulmonary inflammation as the driving force. There was no significant difference between BAL and sputum results.

**Discussion:** CF-ABLE score was further validated as a robust prognostic tool. MMP activity can be used as a biomarker of disease severity. Sputum can be used as a less invasive research matrix, alternative to BAL.

## A5: Trends of use of anti-platelets in Qatar: A retrospective, cross-sectional, a descriptive study from Jan 2015 - Jun 2017

### Iqrah Qurishi, Sara Hussein, Hazem Elewa

#### Qatar University, Qatar University, Doha, Qatar

##### **Correspondence:** Iqrah Qurishi

**Introduction:** Clinical trials have shown the efficacy of new P2Y12 inhibitors (P2Y12i) over clopidogrel in reducing cardiovascular events. Limited studies have investigated the trends of P2Y12i since the approval of prasugrel and ticagrelor with none from the middle east region. The study aims to evaluate the trends of use of P2Y12i and the extent to which clopidogrel was replaced by novel P2Y12i in Qatar.

**Methods:** A descriptive, retrospective, cross-sectional study is performed across Hamad Medical Corporation(HMC) in Qatar. Data were retrieved from the Cerner registry from January 2015 to June 2017. All patients receiving P2Y12i for at least 3 days during the designated period were included. Data was stratified based on the semi-annual period(S1-S5) and by P2Y12i (clopidogrel, ticagrelor, and prasugrel). This study was approved by the HMC and Qatar University review boards.

**Results:** A total of 15,880 patients were included in the study. Most patients were aged between 50-65 and were male. With 15,292 patients in the clopidogrel group, 561 in the ticagrelor and 27 from the prasugrel group. The trend of use across the five-semi-annual blocks ranged from 95.9%-96.9% (S1-S5) for clopidogrel, 3.9%-3.0% (S1-S5) for ticagrelor and 0.2%-0.1% (S1-S5) for prasugrel. These trends remained relatively constant throughout the 5 periods.

**Discussion:** Clopidogrel remained the most commonly used P2Y12i followed by ticagrelor and then prasugrel throughout the study period. The trend changes of the drugs are minor, showing little change in prescribing practice. This raises the question of why newer drugs are not incorporated into practice despite being recommended by international guidelines.

## A6: The effect of quinacrine on cell viability and autophagy induction in U87-MG glioblastoma cells

### Rebecca Arthur, Philip Welsby

#### University of Central Lancashire, Pharmacy and Biomedical Sciences, Preston, UK

##### **Correspondence:** Rebecca Arthur

**Introduction:** Despite current glioblastoma treatments, 90% of patients survive less than two years often due to therapeutic-resistance, hence, there is an ever-growing need for novel therapies. Quinacrine, a former antimalarial drug has had success linked to autophagy in other malignancies. It has a known side effect profile and current drug approval, so it could be re-purposed quickly making it an ideal candidate. We aimed to investigate the effect of quinacrine on cell viability and autophagy induction.

**Methods:** Cell viability and autophagy induction assays were performed to assess cytotoxic activity in U87-MG and SVG-p12 cell lines when compared to cisplatin under normoxic and hypoxic conditions.

**Results:** The treatments reduced cell viability over a 72-hour period in a concentration and time-dependent manner. The effect of quinacrine was not significant when compared to cisplatin. The IC50 values indicated that quinacrine was not affected by oxygen concentration, but cisplatin was, with reduced efficacy in hypoxia. Autophagy assay results suggested that cisplatin significantly induced autophagy in both cell lines. Whereas, quinacrine and rapamycin (the positive control) appeared to not induce autophagy.

**Discussion:** Cell viability results suggested quinacrine has a similar efficacy to cisplatin and therefore has potential as a glioblastoma therapy. The IC50 values indicated that as quinacrine efficacy was unaffected in normoxia and hypoxia that it may be able to overcome therapeutic-resistance as this resistance is associated with hypoxic tumour regions. The autophagy assay results suggested either quinacrine and rapamycin did not induce autophagy, or that the untreated cells had higher autophagy induction due to cellular stress.

## A7: Using YouTube for learning cardiac embryology – what quality of videos are there to view?

### Niveta Ramakrishnan^1^, Jane Holland^2^, Teresa Pawlikowska^2^, Ruth Matthew^2^

#### ^1^Medical Student, Royal College of Surgeons Ireland, Dublin, Ireland; ^2^Royal College of Surgeons Ireland, Department of Anatomy, Dublin, Ireland

##### **Correspondence:** Niveta Ramakrishnan

**Introduction:** Introduction The study of embryology is an integral part of medical curricula, though it is a difficult subject to grasp conceptually. Most medical schools, including RCSI, teach embryology through large group teaching (i.e. lectures) and may provide external links to web-based resources, YouTube videos and textbooks. The use of 2-D visuals, for example animations, allows the student to visualise the structures mentioned in lectures and textbooks, and how their form changes in the fourth dimension – time. Where do students generally find these visuals?

**Methods:** Methods A systematic literature review was performed in PubMed and Embase, to identify any references examining the use of YouTube in teaching or learning cardiac embryology. While an initial 224 references were retrieved for review, and a further 63 references identified through subsequent targeted searches, we found no papers examining this question, and only one other related study examining the quality of YouTube videos concerning gastrointestinal neonatal (and embryological) conditions. Next, YouTube.com was searched using 12 search terms such as “cardiac embryology” and “atrial septation”. A total of 1200 videos were retrieved; some video urls were retrieved under two or more of the search terms (n = 370) and were excluded as “duplicates”. A further 456 videos were excluded under 13 specific criteria including language, non-human or non-cardiac embryology. The remaining 374 videos were then qualitatively evaluated using criteria that Azer et al and Chan et al develop in their respective studies of e-Learning resources. While the qualitative evaluation of the identified YouTube videos is still ongoing, some initial findings and observations may be reported.

**Results:** Results Content control on YouTube, with regard to copyright or explicit imagery, is still imperfect. Five videos identified through our initial searches in June 2018 were subsequently removed form YouTube over the course of the summer for issues including content infringement, and our team also excluded 26 videos from our qualitative analyses, as they were duplicates of other, original videos identified under our search terms. Although we identified 49 videos showed operative procedures or human material, none contained an explicit ethical statement regarding the use of these images. Furthermore, only 10 of these videos included a warning about the nature of the images, such as a graphical advisory or age-restriction.

**Discussion:** Discussion and conclusions To summarise findings to date, while there are certainly videos of use to medical students studying cardiac embryology present on YouTube, intuitive search strategies will also identify many videos with irrelevant content and of variable quality. Some, which claim to be educational, may score very low on the rating scale, while some which have been very simply shot and edited, with minimal resources, are quite excellent.

## A8: Investigating the impact of microRNA inhibition on immune intercellular communication

### Aser Labib^1^, Claire McCoy^2^, Conor Duffy^2^

#### ^1^Medicine, Royal College of Surgeons in Ireland, Dublin, Ireland; ^2^Royal College of Surgeons in Ireland, Molecular and Cellular Therapeutics, Dublin, Ireland

##### **Correspondence:** Aser Labib

**Introduction:** In Multiple Sclerosis (MS), disease progression is associated with a pro-inflammatory macrophage phenotype, including pro-inflammatory exosome secretion. A micro RNA, miR-155 is thought to be involved in the regulation of these pathways, however the mechanism is unclear. Identification miR-155 targets in this secretory pathway will aid in understanding the function of miR-155. Consequently, this could highlight therapeutic targets that may be of clinical significance in patients suffering from MS and other autoimmune conditions.

**Methods:** We stimulated RAW 264.7 cells, a macrophage cell line, with LPS, LPS and IL-10 and IL-10 alone and recorded changes in Rab11fip1 transcription and miR-155 expression vie real time PCR.

**Results:** At 4 hours of stimulation,: Rab11fip1 transcription increased 4.05-fold with LPS and 3.13-fold with LPS and IL-10. miR-155 expression increased 2.51-fold with LPS and 1.36-fold with LPS and IL-10.

**Discussion:** The induction of miR-155 expression by LPS and increase in Rab11fip1 transcription in addition to the relative inhibition of miR-155 and Rab11fip1 when co-stimulated with IL-10 supports a relationship between the gene and the miR-155. This relationship can show that miR-155 regulates Rab11fip1 and can be a method by which miR-155 influences the secretion of exosomes. This property may be of interest in developing novel therapeutics for MS.

## A9: Spinal low-grade gliomas in Canadian children: A retrospective review

### Sukhmani Benipal^1^, S Rod Rassekh^2^

#### ^1^Faculty of Medicine, Royal College of Surgeons in Ireland, Dublin, Ireland; ^2^BC Children's Hospital, Department of Pediatric Hematology/Oncology/BMT, Vancouver, Canada

##### **Correspondence:** Sukhmani Benipal

**Introduction:** Low-grade glioma (LGG) is the most common pediatric central nervous system tumour. Spinal tumours are rare and difficult to treat, with the treatment of spinal LGG (SLGG) being controversial. The Canadian Pediatric Brain Tumour Consortium (CPBTC) undertook a retrospective review of SLGG to primarily identify prognostic factors for patients. We hypothesize that five-year progression free/overall survival of SLGG will be better than published data on intracranial LGG, with Chemotherapy/Radiation therapy showing similar efficacy. And there being a trend towards less aggressive therapy overtime.

**Methods:** With ethics approval, a retrospective chart review was performed. Patients with primary SLGG under 18, treated at BC Children’s Hospital between 1990-2015 were included. Data collected includes date/age at diagnosis, gender, known tumour predisposition syndrome, symptoms, time to imaging, pathology, treatment, post-operative symptoms, neurological recovery, study enrolment, recurrence/progression, treatment of recurrence, and if died of disease. Descriptive statistics were performed.

**Results:** : Results show, 28 subjects with SLGG, 89% are alive. Metastatic disease was seen in 3 (10.7%). Only 32% were able to have a gross total resection (GTR). 57% of subjects had recurrent disease. The most common presenting complaint was pain (64%), followed by weakness (54%) and scoliosis (36%). The majority (54%) had over 6 months of symptoms prior to imaging being performed.

**Discussion:** Children with spinal tumours have good outcomes, despite a low numbers of GTR. Disease recurrence requiring further surgery, chemotherapy/radiation therapy is common. Children with back pain, weakness or scoliosis should have prompt neuroimaging, as there was a delay in diagnosis.

## A10: Risk of QT prolongation and torsades de pointes (TdP) associated with use of SSRIs: Audit on ECG uptake in rural primary care centre in Clare

### Kristin Delcellier^1^, Conor Mcgee^2^, Alison Philips^2^

#### ^1^Graduate Entry Medical School, University of Limerick, Limerick, Ireland; ^2^University of Limerick, Scarriff Medical Centre, Scarriff, Ireland

##### **Correspondence:** Kristin Delcellier

**Introduction:** The aims of the audit were to establish the prevalence of patients on SSRI’s in a rural practice, identify risk factors for QT interval prolongation, and determine ECG adherence according to current guidelines.

**Methods:** A retrospective analysis of the patient database (SOCRATES) at the practice was performed to identify all patients above the age of 16 prescribed an SSRI (Citalopram, Escitalopram, Fluoxetine, Paroxetine and Sertraline) from September 2016 to September 2017. Data was extracted from the identified medical records: sex, age, type of SSRI, history of previously identified cardiac condition, concurrent use of QT prolonging medication, GMS/private patient status and ECG uptake. SPSS was used for data analysis (descriptives and cross-tabs).

**Results:** A total of 258 patients in the practice were being prescribed SSRIs. Risk factors for QT prolongation were cardiac conditions (34.5%), female sex (60.5%), age > 65 (42.2%) and being on other QT prolonging medication (47.7%). ECG monitoring among patients on SSRIs was higher for GMS patients (38.7%) than for private patients (18%). ECG monitoring in patients on SSRIs was best performed overall in patients with cardiac conditions (57.3%).

**Discussion:** It has been identified in recent years that the use of certain SSRIs can lead to QT prolongation. As such, best practice guidelines have been set forth for ECG monitoring of patients with risk-factors, as well as awareness for the other QT prolonging medications taken by patients. The current audit analyzed ECG monitoring of patients on SSRIs between 2016 and 2017, and identified a need for closer monitoring of such patients.

## A11: Assessing the need to provide contraceptive services to women attending addiction services at Cork-Kerry Community Healthcare

### Julia Olioff^1^, Tanya O'Shea^2^, Anna Marie Naughton^3^, Declan O'Brien^2^

#### ^1^Medicine, University College Cork, Cork, Ireland; ^2^Cork-Kerry Community Health, Addiction Service, Cork, Ireland; ^3^HSE Homeless Service, Homeless Service, Cork, Ireland

##### **Correspondence:** Julia Olioff

**Introduction:** Women with substance use disorders who have unintended pregnancies face unique challenges. A common strategy for preventing unintended pregnancies among these women is to increase their use of long acting reversible contraception (LARC). This study assessed the pregnancy history and contraceptive use of women attending Cork-Kerry Community Healthcare for opioid substitution therapy, and their access to contraceptive services. The need for a contraceptive service within the addiction services at Cork-Kerry Community Healthcare was evaluated.

**Methods:** The study utilized a cross-sectional survey administered by healthcare providers to 39 women ages 18-50 attending Cork-Kerry Community Healthcare for opioid substitution therapy. Results were compared to Irish national data on pregnancy history and contraception use.

**Results:** 79% of participants had unintended pregnancies, and 23% had 3 or more unintended pregnancies. Of the participants’ children, 35% lived with their mother, 37% lived in care, and 24% lived with another family member. 31% of participants had never used LARC. 22.5% of participants reported never having received information on pregnancy prevention and 25.6% reported never having received information on STI prevention. 92% of participants reported that they would use a contraceptive service if it were provided within the addiction services at Cork-Kerry Community Healthcare.

**Discussion:** This study highlights the need to increase contraceptive services for women attending addiction services at Cork-Kerry Community Healthcare. Addiction services are ideal locations to also access contraceptive services because service users already attend these clinics frequently for treatment, and thus have continuity of care with healthcare providers.

## A12: Assessment of a stress-reduction intervention for pregnancy-related pelvic girdle pain: a pilot study

### Rachel Howard^1^, Siobhain O'Mahony^2^, Mags Clark-Smith^3^, Ali Khashan^2^, Fergus McCarthy^2^

#### ^1^School of Medicine, University College Cork, Cork, Ireland; ^2^University College Cork, Anatomy and Neuroscience, Cork, Ireland; ^3^Resolving Chronic Pain Clinic, , Kilmacanogue, Ireland

##### **Correspondence:** Rachel Howard

**Introduction:** Pregnancy-related pelvic girdle pain (PPGP) refers to pain in the lumbosacral, sacroiliac and/or symphysis pubis joints. PPGP is present in nearly 20% of pregnancies, and in up to 10% of patients, may persist post-partum for years. The aetiology is poorly understood, but stress during pregnancy is predictive for persistent post-partum pain. Evidence for the efficacy of current treatments has been limiting and conflicting, and treatment efforts often unsuccessful. This study aims to assess the effectiveness of a stress-reducing intervention on pain symptoms associated with PPGP.

**Methods:** 15 women with PPGP were recruited from Cork University Maternity Hospital (CUMH). Participants received two, one-hour sessions; information on the stress response, gentle exercises, and confidence-building counselling were provided. Before and after each session, salivary samples were obtained and cortisol was quantified with ELISA. Participants completed the Roland Morris Disability Questionnaire, Cohen Perceived Stress Scale, Stait Trait Anxiety Questionnaire, and the number of steps possible in 60 seconds was recorded.

**Results:** 15 participants completed the first intervention session, of which 7 completed both sessions. The questionnaire results and steps per minute indicated the intervention led to a significant improvement in anxiety, pain, stress and mobility. A significant decrease in salivary cortisol was measured after the two sessions, compared to the initial baseline measurement.

**Discussion:** The potential benefit of a stress-reducing intervention for women experiencing PPGP has been illustrated by this study, through reducing pain, stress, and anxiety while concurrently improving mobility. This data will support a feasibility trial study, followed by a multi-centre clinical trial.

## A13: Proteomic analysis of MYCN in neuroblastoma

### Sian Roberts-Walsh^1^, Melinda Halasz^2^

#### ^1^School of Medicine, University College Dublin, Dublin, Ireland; ^2^University College Dublin, Systems Biology Ireland and School of Medicine, Dublin, Ireland

##### **Correspondence:** Sian Roberts-Walsh

**Introduction:** Neuroblastoma is an embryonal tumour originating from the peripheral sympathetic nervous system. MYCN amplification (seen in about 22% of cases), associated with poor patient survival, is one of the strongest determinants of clinical outcome. This project aimed to characterise the MYCN interactome in the BE(2)-C MYCN-amplified neuroblastoma cell line, and identify novel targets for the therapy of neuroblastoma.

**Methods:** Initially, protein-protein interaction partners of MYCN were identified by MYCN co-immunoprecipitation coupled to mass spectrometry. Analysis of this data by String and Ingenuity Pathway Analysis (IPA) software determined the most altered pathways associated with MYCN amplification, one of which is mTOR. The effect of an mTOR inhibitor, Rapamycin, on BE(2)-C cells alone or in combination with aurora kinase inhibitors Tozasertib and Alisertib was determined using microscopic phenotypic observation and cell death analysis by flow cytometry.

**Results:** 96 proteins were identified as MYCN interactors in BE(2)-C cells. IPA identified that the MYCN binding proteins participate in mTOR signalling and regulation of the cell cycle. All tested mono- and co-treatments decreased proliferation and increased cell death compared to the control. Aurora kinase inhibitors increased differentiation more than Rapamycin alone and co-treatments more than mono-treatments. Alisertib and Rapamycin co-treatment(48.75%) increased cell death compared to Alisertib(36.6%) or Rapamycin(10.65%) alone while there was no marked difference between Toszasertib mono- and co-treatments.

**Discussion:** Targeting signalling pathways which orchestrate MYCN’s oncogenic functions is an effective way to inhibit neuroblastoma growth. Further validation in pre-clinical trials is necessary to verify the synergistic potential of these new drug combinations.

## A14: Assessing the molecular alterations induced by lncRNA SATB2-AS1 in colorectal cancer

### Caitlyn Loo^1^, Sudipto Das^2^, Kian Heart^3^, Sara Terer^2^

#### ^1^School of Medicine, Royal College of Surgeons in Ireland, Dublin, Ireland; ^2^Department of Molecular and Cellular Therapeutics, Royal College of Surgeons in Ireland, Dublin, Ireland; ^3^Trinity College Dublin, Dublin, Ireland

##### **Correspondence:** Caitlyn Loo

**Introduction:** lncRNAs have been widely shown to function as both tumour suppressors and oncogenes across various cancer types. Studies conducted in The Royal College of Surgeons in Ireland on lncRNA SATB2-AS1, the antisense to oncogenic protein-coding gene SATB2, has shown a reduction in colon cancer cell proliferation and colony forming ability in SATB2-AS1 knockdown samples, achieved using RNA interference. Additionally, there was increase in SATB2-AS1 expression from primary tumour to liver metastasis. This was substantiated by the only paper on SATB2-AS1. In light of these, understanding the mechanistic action of SATB2-AS1 was required to evaluate its potential as an anti-proliferative target.

**Methods:** SATB2-AS1 was silenced using two independent siRNAs and transfected into SW480 colon cancer cell lines. RNA was extracted, isolated and purified at 96 and 120 hour post-transfection. cDNA was synthesised and used to determine the relative target gene expression of various markers of inflammation and epigenetic regulators such as IL-1b, TNF-a, VEGF-A, IFN-g, IL-18, Caspase-1, Caspase-4, DNMT1, DNMT3A, DNMT3B and EZH2, through quantitative real time polymerase chain reaction (qRT-PCR).

**Results:** qRT-PCR revealed an upregulation of IL-1b and TNF-a and a downregulation of IFN-g, DNMT1 and DNMT3A, suggesting that SATB2-AS1 knockdown leads to transcriptional alterations of inflammatory cytokines and downregulation of DNA methyltransferases.

**Discussion:** These results have given insight into the oncogenic mechanism of SATB2-AS1, which could be acting alongside SATB2, as SATB2-AS1 knockdown seems to have similar effects on inflammatory cytokines when compared to results from SATB2 knockdown. However, as these results are preliminary, more repetitions are needed to examine their validity.

## A15: Establishing patient derived xenografts for clinical application in the management of colorectal cancer

### Lorraine Chu^1^, Tony Mok^2^

#### ^1^Medicine, Royal College of Surgeons in Ireland, Dublin, Ireland; ^2^The Chinese University of Hong Kong, Clinical Oncology, Hong Kong, Hong Kong

##### **Correspondence:** Lorraine Chu

**Introduction:** Cancer is one of the leading causes of death worldwide. Much research has gone into prevention, early diagnosis and development of anti-cancer treatments. However, there is little understanding of proliferation and resistance in cancer cells as well as each individual patient’s response to anticancer agents. Patient derived xenograft (PDX) is the engrafting of a patient’s tumour cells into immunodeficient mice, while maintaining the histopathological, genetic and phenotypic integrity of the original tumour. By doing this, we are able to establish clinically predictive models of human cancers to better personalise treatment options.

**Methods:** Primary tumours are excised from patient and transplanted into immunodeficient mice. Tumours >15mm3 in mice are then excised and transplanted to the next generation. Tumour cells from different generations of immunodeficient mice are stained with H&E and immunohistochemical markers (EPCAM, CDX2). Cell morphology and molecular differences are evaluated between the different generations of the PDX model.

**Results:** Results show that the PDX models for both patients were successfully established. Histopathology and immunohistochemistry of the tumours showed similarities between the patient’s original tumour and the subsequent generations in the PDX model. This demonstrates that the tumour grafts conserved the genotypic integrity of the patient’s tumour.

**Discussion:** PDX models can be used in basic cancer research involving characterisation, tumorigenesis, and metastasis. Using the PDX model as a cancer avatar for the patient, we are able to do preclinical and co-clinical studies on drug screening, future drug development and biomarker discovery.

## A16: Creating a novel virtual/electronic whiteboard (vWB) – A new multidisciplinary tool in coordinating care for patients with dementia

### Allyson Coopersmith^1^, Pieter Jugovic^2^, Shaghayegh Rezaie^2^

#### ^1^School of Medicine & Department of Family and Community Medicine, Royal College of Surgeons & University of Toronto, Dublin, Ireland; ^2^University of Toronto & Michael Garron Hospital, Department of Complex Continuing Care & Department of Family and Community Medicine, Toronto, Canada

##### **Correspondence:** Allyson Coopersmith

**Introduction:** There are barriers within hospitalised care that prevent the transition of inpatients with dementia to best possible dispositions. We hope that the creation of the vWB and the data being collected through the vWB and surveys, as well as the feedback from the DCCC staff, will help us enhance the vWB tool and determine the inhibiting factors that provoke these delays.

**Methods:** We attended rounds in the DCCC, projected the vWB onto a screen, and recorded relevant data for each patient onto the database. To narrow our focus on the most important variables, we created a needs assessment survey and conducted staff interviews. Relevant clinical literature was reviewed on the Health Quality Ontario website. This information, along with the results from the surveys and interviews helped to resolve our focus on the variables that can be collected on the vWB.

**Results:** Staff interviews and surveys revealed variables measuring behaviour monitoring, length of stay, and disposition planning were of greatest concern.

**Discussion:** Most dementia patients have extensive waiting periods for admission to long-term care that exceed an anticipated time frame. Demand for beds in chronic care facilities exceed availability. Many patients with stable dementia cannot be transitioned as a result. External behavioural supports, who come to the DCCC to help manage patient behaviours, are in short supply. To this end, profiles of behaviours including their types, frequencies, and disruptiveness were variables chosen by participants. We hope the creation of the vWB will help our dementia patients have an easier transition to their final destination.

## A17: Compliance with prescribing guidelines for acute low back pain in a general practice

### Dean Simonsky^1^, Paraic Meaney^2^, Marese Mannion^2^, Fergus Glynn^2^

#### ^1^Graduate Entry Medicine, University of Limerick, Limerick, Ireland; ^2^Corofin Medical Centre, Corofin Medical Centre, Corofin, Co. Clare, Ireland

##### **Correspondence:** Dean Simonsky

**Introduction:** Estimates suggest that low back pain affects up to 84% of the adult population and cost the Irish government €300 million in disability payments in 2002. The American College of Physicians (ACP) clinical guideline (2017) recommends nonpharmacologic (NP) treatment as a first-line management for acute & subacute low back pain (ALBP); skeletal muscle relaxants (SMRs) and nonsteroidal anti-inflammatory medications (NSAIDs) are recommended as second-line treatment. Management of acute presentations can reduce the risk of progression to a chronic disease state. This study assessed the types of intervention provided to patients with ALBP, compared ALBP management among General Medical Services Scheme (GMS) and non-GMS patients, and assessed whether ALBP management was consistent with ACP guidelines.

**Methods:** The patient database was searched for adults who presented to Corofin Medical Centre in 2017 with new-onset, non-radicular, low back pain. Eighty-two patients satisfied the inclusion criteria and comprise the study population.

**Results:** Fifty percent of patients received NP treatment, 15% received opioids, and no patients received SMRs. NP-only and pharmacologic (PH) management rates were similar among GMS and non-GMS patients. NSAIDs were prescribed more frequently to GMS patients while opioids were prescribed more frequently to non-GMS patients.

**Discussion:** Only 50% of patients received NP therapy as first-line management yet this should be considered for all patients before PH management. SMRs should be considered in addition to NSAIDs when NP management proves inadequate. A reluctance toward opioid prescription should be adopted as opioids are not recommended in the ACP guideline.

## A18: The role of lymphoscintigraphy in sentinel lymph node biopsy for breast cancer: An audit of 100 lymphoscintigrams in a tertiary Irish centre

### Ashraf Alzahrani^1^, Arnold Hill^2^

#### ^1^Department of surgery, Royal College of Surgeons in Ireland, Dublin, Ireland; ^2^Royal College of Surgeons in Ireland, Department of surgery, Dublin, Ireland

##### **Correspondence:** Ashraf Alzahrani

**Introduction:** Sentinel lymph node biopsy (SLNB) has frequently been demonstrated to accurately stage axillary lymph nodes in breast cancer. When performed, preoperative lymphoscintigraphy (LSG) is commonly used as a complementary modality to enhance the identification of sentinel lymph nodes (SLNs). However, the clinical significance of LSG remains debated.

**Methods:** 100 breast and axillary lymph nodal status were evaluated retrospectively. Data was obtained from Beaumont Hospital online databases and medical records. 97 patients were assessed by LSG using radioisotope and subsequently by SLNB. Three patients had bilateral nodal assessments. Results of the LSG, SLNB and histology report were analysed and compared on Microsoft Excel.

**Results:** LSG identified SLNs in 90 cases (90%). SLNs were identified surgically in all cases with a negative LSG (n=10). The mean number of nodes identified by modality was 1.39 by LSG, 1.36 by surgery, and 2.4 by histology. 27 cases were positive for nodal metastases. The number of nodes on LSG was identical to surgery and histology in only 49% and 35% of cases, respectively.

**Discussion:** LSG does not have a significant role in the identification of SLNs. Moreover, the result of LSG does not accurately reflect the number of nodes identified surgically or histologically.

## A19: Water, sanitation and hygiene and the risk of malnutrition in Indian children: Recent evidences from NFHS4

### Nishant Raman^1^, Aditya Rana^2^, Puja Dudeja^2^, V Sashindran^2^, Varsha Renjit^2^

#### ^1^Graduate Wing, Armed Forces Medical College, Pune, India; ^2^Armed Forces Medical College, Graduate Wing, Pune, India

##### **Correspondence:** Nishant Raman

**Introduction:** It is increasingly apparent that water, sanitation, and hygiene are important determinants of malnutrition in children besides that of food availability. This study draws an association of water, sanitation and hygiene (WaSH) as potential risk factors for stunting, wasting and growth faltering among 2-5-year-old Indian children.

**Methods:** Nationally representative data on 90,867 children from Indian National Family Health Survey NFHS-4 was analysed for determinants of malnutrition based on UNICEF conceptual framework. Statistical analysis involved descriptive and univariate regression to select 20 variables comprising of maternal, child and environmental factors to be included in the final multivariable models- each for stunting, underweight and wasting and multiple logistic regression to determine associations.

**Results:** With a 15-36% reduction in prevalence of different forms of malnutrition, access to toilets and safe disposal of child's feces emerge as pertinent determinants of malnutrition in children, sanitation also being associated with lowering of odds of stunting by 11%, underweight by 10% and wasting by 7%. Adjusting for potential confounders, water-related factors were however not significantly associated with risk of malnutrition. No interaction was observed between water and sanitation.

**Discussion:** In the given setting, among all wash indices, sanitation emerged to be the single modifiable entity associated with significant reduction in the risk of both acute and chronic malnutrition with greater impact compared to water. Concluding, the present study establishes the magnitude of impact effective integration and robust implementation of WaSH interventions with other nutrition specific measures can have in national programmes for primary level prevention and ultimate elimination of malnutrition.

## A20: Novel blood biomarkers for risk stratification, prognosis, and mechanistic insight in children with severe TBI

### Alexandr Magder^1^, Jamie Hutchison^2^

#### ^1^Critical Care Medicine, The Hospital for Sick Children, Toronto, Canada; ^2^The Hospital for Sick Children, Critical Care Medicine, Toronto, Canada

##### **Correspondence:** Alexandr Magder

**Introduction:** Traumatic Brain Injury (TBI) is the most common cause of acquired disability in children. The molecular mechanisms are not well understood and acutely clinicians are often unable to prognosticate. We hypothesized that low abundance proteins, measured in blood, would relate to mechanisms of secondary brain injury and be associated with long-term global neurological outcome.

**Methods:** Demographic, injury severity and physiologic data was collected from 210 pediatric patients. 112 (53%) of these patients had severe TBI defined as a Glasgow coma scale (GCS) of ≤ 8 on admission. We then chose 8 of these children with blood samples drawn within 24 hours of TBI and an unfavorable outcome (severe disability or death) at 6 months following TBI and matched each using GCS, age and sex to 8 other children with a favorable outcome (normal or mild disability) at 6 months post-TBI. Low abundance proteins were measured using untargeted mass spectroscopy. Scaffold Q+ proteomic software was used to compare semi-qualitative differences in serum concentrations of identified proteins focusing on statistical differences between the two experimental groups.

**Results:** Twenty-four proteins were statistically different between the favourable and unfavourable group, including metabolic, anti-oxidant, and vasoactive proteins. Six were consistently found in poor outcome patients and one unfavourable in positive outcomes, which corroborated mechanistic evidence in previous literature.

**Discussion:** These insights may lead to new molecular therapies and have potential to greatly improve clinical decision making and risk stratification models for clinical trials.

## A21: Elucidating the role of FKBPL in the IL-6/STAT3 signaling pathway

### Uchechukwu Alanza^1^, Tracy Robson^2^, Gillian Moore^2^, Evan Woods^2^, Stephanie Annett^2^

#### ^1^Molecular and Cellular Therapeutics, Royal College of Surgeons in Ireland, Dublin, Ireland; ^2^Royal College of Surgeons in Ireland, Molecular and Cellular Therapeutics, Dublin, Ireland

##### **Correspondence:** Uchechukwu Alanza

**Introduction:** FKBPL is an anti-cancer protein that targets CD44 receptors to inhibit cancer angiogenesis and stemness. Based on the active region of FKBPL, a biological drug was developed and has successfully completed Phase I clinical trials (EudraCT No: 2014-001175-31). STAT3 is known to interact with CD44 and its dysregulation is associated with increased cancer tumourigenesis, metastasis and stemness. We performed a knockdown of FKBPL gene expression in ovarian (OVCAR3) and breast (MDA-MB-231) cancer cell lines to investigate the effect this had on the IL-6/STAT3 signaling pathway.

**Methods:** OVCAR3 and MDA-MB-231 cells were transfected with scrambled or targeted FKBPL siRNA for 48 hours. Following RNA extraction, cDNA was analysed using an RT2 PCR Profiler Array containing 84 genes related to IL-6/STAT3 signaling. A minimum threshold of 2-fold was chosen as a cut-off to identify genes of interest. Candidate genes chosen for validation were measured by RT-qPCR (N=4).

**Results:** CCL4, CXCL10 and TNFRSF1B were upregulated while PIM1 and LIFR were downregulated in OVCAR3 following FKBPL siRNA knockdown. JAK3, IL6R, EGFR, BCL2, IL4, CCL3 and TLR4 were upregulated while IL6 and IL11 were downregulated in MDA-MB-231 following FKBPL knockdown. 5 genes from the MDA-MB-231 array have been re-validated by qPCR (N=4). EGFR (1.89, p<0.01), BCL2 (1.96-fold, p<0.01) and TLR4 (1.39-fold, p<0.05) were upregulated while IL6 (-4.16-fold, p<0.001) and IL11 (-1.89-fold, p<0.05) were downregulated following FKBPL knockdown.

**Discussion:** This data highlights a novel interaction between FKBPL and the IL-6/STAT3 signaling pathway. Thus, FKBPL may elicit its known anti-cancer activity through a modulation of the IL6/STAT3 signaling pathway.

## A22: Profiling high endothelial venules and other blood vessels in cancer immunotherapy

### Eshkeerat Kaur^1^, Ann Ager^2^, Ruban Durairaj^2^

#### ^1^Medicine, Royal College of Surgeons, Dublin, Ireland; ^2^Cardiff University, Institute of Infection and Immunity, Cardiff, United Kingdom

##### **Correspondence:** Eshkeerat Kaur

**Introduction:** High Endothelial Venules (HEVs) are specialized blood vessels that support the migration of naïve lymphocytes from the bloodstream into secondary lymphoid organs. A regulated sequence of adhesive interactions are mediated by different adhesion molecules that allow binding of lymphocytes to surface of HEVs and are critical for lymphocyte entry into lymph nodes. HEVs develop in non-lymphoid organs during chronic inflammation and are associated with increased lymphocyte infiltration. Recently, HEVs have been observed in solid, vascularized cancers such as breast cancer and melanoma and their presence is correlated with improved patient outcomes. It is proposed that newly formed HEV promote anti-cancer immunity by recruiting lymphocytes into the cancer. The aim of this project is to determine whether HEVs develop in mouse models of cancer immunotherapy

**Methods:** Important murine HEV markers (e.g. GlyCAM-1) were selected and the appropriate primers designed. RNA was extracted from untreated mouse lymph nodes and spleens positive and negative controls. Isolated RNA was analysed for impurities before cDNA synthesis was carried out. RT-PCR was used to analyse cDNA for the selected genes and. Beta-2-microglobulin was used as control.

**Results:** The analysis of the RT-PCR showed an increased amplification for GLYCAM-1 cDNA, specifically in the lymph node samples, as HEVs not present in the spleen. However, significant results were not seen for other HEV markers or endothelial markers.

**Discussion:** In conclusion, GLYCAM-1 was considered an important marker for detecting HEVs. The next step is to analyse tumours from mice that have had immunotherapy.

## A23: Implementing a post-discharge prophylactic venous thromboembolism protocol in cancer patients undergoing colorectal surgery at St. Michael’s Hospital

### Caitlin Gibson^1^, Ori Rotstein^2^, Vera Dounaevskaia^2^, Sandra de Montbrun^2^

#### ^1^Medicine, RCSI, Dublin, Ireland; ^2^University of Toronto/St. Michael's Hospital, Department of Surgery, Toronto, Canada

##### **Correspondence:** Caitlin Gibson

**Introduction:** Post-operative VTE remains one of the leading complications and causes of mortality in colorectal surgery patients with cancer. While there is evidence that continuation of VTE prophylaxis beyond the hospital stay can reduce overall incidence of VTE in select groups of surgical patients, local hospital guidelines for prescribing post-discharge prophylactic medication to prevent VTE vary in practice. The aim of this project was to develop an evidence-based protocol for post-discharge VTE prophylaxis on colorectal cancer patients undergoing surgical resection at St. Michael’s Hospital, Toronto.

**Methods:** The project consisted of a literature review surrounding VTE prophylaxis in cancer patients, a chart review of 100 cancer patients undergoing colorectal surgery to determine compliance with best practice, and possible implementation of a new thromboprophylaxis protocol.

**Results:** Current guidelines recommend 30-day post-operative thromboprophylaxis for cancer patients undergoing major abdominal or pelvic surgery. After review of the 100 cases, 21 cases were excluded from analysis because they did not have a laparotomy or laparoscopic procedure or they were on anticoagulation therapy at time of/during admission. Only 2 of the 79 remaining cases (2.53%) were prescribed post-discharge thromboprophylaxis.

**Discussion:** This demonstrates a gap in the use of recommended guidelines for the prevention of VTE in colorectal resections for cancer patients. A protocol for implementation of post-discharge VTE prophylaxis in cancer patients undergoing colorectal surgery at St. Michael’s Hospital, Toronto was developed and is in the beginning stages of implementation. This knowledge translation approach will serve to improve outcomes in patients undergoing colorectal cancer surgery at the Institution.

## A24: Deprescribing of long-acting sulfonylureas at the Michael Garron Hospital

### Colum Horan^1^, John Abrahamson^2^

#### ^1^School of Medicine, Royal College of Surgeons in Ireland, Dublin, Ireland; ^2^Michael Garron Hospital, Department of Medicine, Toronto, Canada

##### **Correspondence:** Colum Horan

**Introduction:** Substantial evidence justifies great caution in the use of long-acting sulfonylureas in the treatment of Diabetes Mellitus, in particular amongst frail patients. Deprescribing is the systematic discontinuing of drugs where existing or potential harms outweigh existing or potential benefits. This study sought to identify cases of long-acting sulfonylurea use at a large, urban, community teaching hospital and utilise evidence-based deprescribing tools to rectify cases where risks outweighed benefits.

**Methods:** During August 2018, all patients admitted to MGH between July 1st 2017 and August 1st 2018 and prescribed Glyburide/Glibenclamide were identified. These were all followed up to ascertain their current medication regimen. Following consultations guided by deprescribng guidelines, individualised deprescribing recommendations were either carried out directly by project team physicians or forwarded to patients' family doctor and community pharmacy. Secondary process measures looking at deprescribing behaviour in MGH were also analysed.

**Results:** 30 patients were identified as being on Glyburide/Glibenclamide during the studied period and eligible for follow up. Of these, 17 (57%) had been appropriately deprescribed prior to the study. All 13 remaining patients were provided with a deprescibing recommendation, of which 4 were carried out by project team physicians and 9 were forwarded to patients' primary physician. Among reviewed patients, no readmissions were attributed to deprescribing recommendations made.

**Discussion:** Deprescribing initiatives focusing on a specific drug or drug class in a community hospital are feasible. A supportive culture, collaboration with Pharmacist colleagues and EMR are key enablers. Reviewing physicians’ decisions to proactively deprescribe may highlight other levers to enhance good deprescribing behaviours.

## A25: Correlation between syndromic surveillance data and rainfall in the Caribbean using endemic channels

### Sumara Jaimungal^1^, Avery Hinds^2^

#### ^1^Medicine, RCSI, Dublin, Ireland; ^2^Caribbean Public Health Authority, Communicable Disease and Emergency Response, St. James, Trinidad and Tobago

##### **Correspondence:** Sumara Jaimungal

**Introduction:** Many diseases endemic to Caribbean countries are monitored regionally via syndromic surveillance. Endemic channels (EC) are being used to supplement EARS (early aberration reporting system) as a means of differentiating anticipated seasonal influxes from true outbreaks in order to be more efficient at in response coordination. The aim of this study is to determine, graphically using endemic channels, any correlation between with the number of cases of syndromes reported and rainfall.

**Methods:** Monthly rainfall data was collected from 13 Caribbean member states (CMS) for the period 2011-2017. This was compared with monthly averages of cases for each of the 8 years, for 3 defined syndromes: fever and respiratory; gastroenteritis; undifferentiated fever from the 13 CMS. ECs were constructed utilizing the geometric mean method. Involved using population data for each year to find rates per month then a logarithmic transformation to find plot values. 8-year rainfall averaged per month was then superimposed on each EC.

**Results:** Gastroenteritis had an inverse relationship with rainfall. Fever and respiratory, as well as undifferentiated fever, did not have any clear relationship with rainfall.

**Discussion:** These findings suggest that during monthly periods of heavy rainfall, reported cases of gastroenteritis are lower than during periods of decreased rainfall. Investigations into food consumption behaviour during periods of decreased rainfall will be useful in determining how the transmission is increased. This information will be valuable in establishing public health campaigns aimed at educating the population in gastroenteritis mitigation strategies as a means of decreasing the burden of the disease.

## A26: SGK3 as a mediator of endocrine resistance in aromatase inhibitor treated breast cancers

### Tareq Andijani^1^, Marie McIlroy^2^, Rachel Bleach^2^

#### ^1^School of Medicine, RCSI, Dublin, Ireland; ^2^RCSI Endocrine Oncology Research Group, Department of Surgery, Dublin, Ireland

##### **Correspondence:** Tareq Andijani

**Introduction:** The steroid hormones estrogen and androgen play a vital role in cell growth and death. Serum and glucocorticoid regulated kinase 3 (SGK3) is a target gene of both estrogen and androgen receptors. We continued on the work that has been previously done in uncovering the relationship between ERa, AR and SGK3 in breast cancer treatment.

**Methods:** First, Human breast cancer cell lines (MCF7 Aro, MCF7, LetR, ZR75.1 and ZRR) were cultured to evaluate the effect of steroid hormone on SGK3 expression. Next, RNA extraction was performed with Qiagen Rneasy column protocol (Exception was made in step 14). Then, Western blotting protocol was applied. Finally, cell proliferation assay was measured by MTS.

**Results:** AI-sensitive cells had higher levels of SGK3 expression than the resistant except MCF7Aro, which showed almost no expression. All treatments in this experiment affected LetR cell survival and growth with estradiol (E2) having the greatest effect on the cell line.

**Discussion:** Increased quantity of SGK3 in AI-resistant cells was expected. However, our results demonstrated a decrease in protein levels. This could be due to numerous reasons. For example, we used translation to quantify the levels of SGK3 rather than transcription. Steroids showed a positive impact on LetR proliferation. However, due to the three days limitation we cannot predict the response after that time frame.

## A27: Insulin Prescribing in an Acute Hospital Setting : A Point Prevalence Audit

### Ola Michalec^1*^, Nikki Cliffe^1*^, Bernie Love^2^, Kate Hourigan^2 ,^Seamus Sreenan^1^

#### ^1^Endocrinology, RCSI, Dublin, Ireland; ^2^Connolly Hospital Blanchardstown, Pharmacy, Dublin, Ireland

##### **Correspondence:** Ola Michalec; Nikki Cliffe

*These authors contributed equally to this work

**Introduction:** Medication errors are a not uncommon and potentially preventable cause of adverse outcomes in hospitalised patients. Insulin is a high risk medication with the potential to cause serious harm if not administered correctly. The aim of this study was to examine current insulin prescribing practices in Connolly Hospital Blanchardstown (CHB) with the intention of developing a quality improvement plan should prescribing errors be identified.

**Methods:** A hospital-wide point-prevalence audit involving all patients in the hospital treated with insulin was conducted over a one week period. This involved reviewing the medication kardex and bedside folder of patients on all types of insulin including: basal, bolus and mixed insulins, sliding scale and intravenous insulin. A number of measures were assessed relative to the expected prescribing standards, including the use of brand name, whether the dose was stated, the use of “units”.

**Results:** During one week, a total of 232 patients were captured across 12 wards with 18 patients identified receiving insulin (8%). A total of 58 errors were identified among the 41 insulin prescriptions, with an average of 1.4 errors per prescription. The most frequent error involved the lack of documentation on the main kardex that additional insulin prescriptions (sliding scale (sc), IV) were in place.

**Discussion:** Analysis of the insulin prescribing suggested that prescribing errors are common. The reasons for the errors are probably complex but a number of system failures could have contributed. An insulin-specific kardex will be designed to reduce the frequency of such errors.

## A28: New method an abdominal advancement flap of combined breast reconstruction

### Júlia Bartková^1^, Martin Boháč^2^

#### ^1^Department of Plastic, Reconstruction and Aesthetic Surgery, Comenius University, Bratislava, Slovakia; ^2^Comenius University, Department of Plastic, Reconstruction and Aesthetic Surgery, Bratislava, Slovakia

##### **Correspondence:** Júlia Bartková

**Introduction:** An Abdominal advancement flap is often used as one of methods of the breast reconstruction with prosthetic materials and dermal matrix after radical mastectomy. We modified the old method of abdominal advancement flap and compared the patients treated with classic and new method.

**Methods:** An abdominal advancement flap is a flap that is pulled up using the skin and the subcutaneous tissue that was under the original inframammary fold and creates the shape of the lower part of the breast by making a neo-inframammary fold. In this new modified method the fixation of the mobilized skin is realized by using a long-term absorbable material installed into the subcutaneous region within the range of the footprint of the new breast which is fixed transpectorally by specialized skeletonized needles. We monitored and compared two groups of patients. In the first reference group of 11 patients during the breast reconstruction was used expander/implant and acellular dermal matrix. In the second group of 11 patients was used besides standard completions also new method of abdominal advancement flap.

**Results:** Using this modified method of abdominal advancement flap, we discovered its relation with initial expansion volume which was 44 percent bigger than at classical method. In the first group of pacients we registered three post-operative complications and in the second group, we noticed two complications.

**Discussion:** This method has more aesthetic and functional effect with the minimal morbidity of the donor site compared to the „conventional method“. This method leads to aesthetically better formed lower part of new breast.

## A29: An observational study of the utility of BRAF and MEK combination therapy in metastatic melanoma in a regional cancer centre

### Fionnuala Crowley^1^, Derek Power^2^

#### ^1^Medical Oncology, University College Cork, Cork, Ireland; ^2^University College Cork, Mercy University hospital, Cork University Hospital, Medical Oncology, Cork, Ireland

##### **Correspondence:** Fionnuala Crowley

**Introduction:** Trametinib (MEK inhibitor) combined with Dabrafenib (BRAF inhibitor), targeted therapy for BRAF mutated metastatic melanoma, was introduced in Ireland in 2012. The clinical outcomes of these drugs have not been evaluated in an Irish population. The relationship between toxicity and outcome has to date not been studied.

**Methods:** A retrospective chart review of all patients (n=41), treated with dabrafenib alone or in combination with trametinib from May 2012 to September 2018 was carried out. Clinicopathologic variables were recorded. The clinical course of all patients was examined in detail. Treatment outcomes were measured using progression free survival (PFS), overall survival (OS) and objective response rates (ORR).

**Results:** 39% of patients experienced grade 3 or greater of a side effect, necessitating a dose reduction. The median PFS of patients who experienced side effects was 12.29 months (95% CI 4.045 to 20.535) versus 5 months(95% CI 2.215 to 7.785) in patients who didn’t experience side effects (p=0.003). The median PFS for patients who had their dose reduced due to side effects was 16.55 months (95% CI 0.426 to 32.674) versus 5.520 (95% CI 3.972 to 7.068) in those whose doses remained the same (p=0.024). The ORR among all patients was 82.9%. When patients with brain involvement (n=12) were removed, the median PFS was 12.35 months (95% CI 0.42 to 24.280) and median OS was 18.19 months (95% CI 13.412 to 22.968).

**Discussion:** The median OS of 18.19 months in patients without brain metastases is lower than the clinical trials median of 25.6 months. This is likely due to our heterogenous population. Our results highlight a statistically significant relationship between toxicity and PFS. A similar situation has been reported with immunotherapy. These findings may impact clinical practice.

## A30: Risk factors, comorbidity, and complications of clostridium difficile infection: A study of patients who died in the course of CDI

### Mateusz Michalak^1^, Mateusz Michalak^2^, Marcin Fedewicz^2^

#### ^1^Faculty of Medicine, Jagiellonian University Medical College, Krakow, Polska; ^2^Jagiellonian University Medical College, Faculty of Medicine, Krakow, Polska

##### **Correspondence:** Mateusz Michalak

**Introduction:** Clostridium difficile (C.diff.) is a Gram-positive, anaerobic bacillus, widely spread in the human environment. In the last decade, the incidence and severity of Clostridium difficile infection (CDI) have been increasing, making this particular disease one of the most significant nosocomial infections. Notable risk factors include antibiotic use, advanced age, previous hospitalization. The patients often present with multiple comorbidities, which makes management challenging.

**Methods:** The study analysed 154 patients (mean age 77) who died diagnosed with CDI in the University Hospital in Krakow, Poland. The focus was on the complication rate, factoring in comorbidities, risk factors and antecedent antibiotic treatment type. Data was collected using standardised forms and statistical analysis was used where applicable.

**Results:** All patients had pre-existing risk factors for CDI in some form. 82,5% (127 out of 154) patients had been treated with antibiotics before developing CDI. Almost half (47%) had been treated with an immunosuppressant, most commonly steroidal. Among the most common pre-existing conditions was diabetes (32%, 49 out of 154 patients). Complications recorded in medical documentation included a high rate of pneumonia (19,5%, 30 patients), AKI (5,8%, 9 patients) and numerous others.

**Discussion:** The epidemiological data of patients who died in the course of CDI paints a picture of a fragile, aged population with a staggering rate of risk factors for CDI, numerous comorbidities and complications. The high rate of pneumonia could be particularly worrying for patients such as those and more emphasis should be put on its prevention and fast diagnosis.

## A31: Retrospective study of competing risk model in screening for preeclampsia at 11-13 gestational weeks

### Sorana Manuca^1^, Dragos Nemescu^2^

#### ^1^Medicine, Grigore T. Popa University of Medicine and Pharmacy, Iasi, Romania; ^2^Grigore T. Popa University of Medicine and Pharmacy , Clinical Obstetrics and Gynecology Hospital Cuza Voda, Obstetrics & Gynecology, Iasi, Romania

##### **Correspondence:** Sorana Manuca

**Introduction:** Preeclampsia (PE) affects about 2-5% of pregnancies and is a major cause of maternal and perinatal morbitidy and mortality. Our aim was to study the risk distribution of PE calculated in our local population at 11-13+6 gestational weeks by means of the FMF (Fetal Medicine Foundation) algorithm which combines a priori risk from maternal characteristics with uterine artery pulsatility index (UtA-P), serum pregnancy-associated plasma protein-A (PAPP-A), placental growth factor (PlGF) and mean arterial pressure (MAP).

**Methods:** Data of 1037 singleton pregnancies at 11+0 to 13+6 weeks’ gestation in women who underwent aneuplodiy screening at our department from December 2009 to October 2017 was analyzed using Astraia and SPSS. Maternal risk factors were recorded, MAP and UtA-PI were expressed as multiples of the median (MoM) and log transformed to make their distribution Gaussian. Twins, major anomalies, aneuploidies and incomplete data were excluded.

**Results:** Out of 124 (14%) nulliparas with high risk (>1/100) for preeclampsia before 37 weeks, we had 94 (75%) available outcomes. These included 8 (9.5%) with preeclampsia, 17 (18%) with gestational hypertension and 5 (6.4%) with fetal growth restriction. From 732 (85.6%) cases with low risk (<1/100) for preeclampsia, we had 357 (49%) available outcomes with 2 cases of severe PE. The risk for PE before 34 weeks was >1/100 in 101 (9.7%) cases and ≤1/100 in 936 cases (90.3%).

**Discussion:** Screening by means of FMF algorithm yielded better results at identifying the risk groups in our population than screening by maternal factors alone.

## A32: The expression of cannabinoid proteins paths in mammalian tissue

### Piotr Przybycień^1^, Piotr Przybycień^2^

#### ^1^Faculty of Medicine, Jagiellonian University Medical College, Krakow, Poland; ^2^Jagiellonian University Medical College, Faculty of Medicine, Krakow, Poland

##### **Correspondence:** Piotr Przybycień

**Introduction:** The endocannabinoid system (ECS) is a natural system composed of endocannabinoids and their receptors. It is disseminated throughout the mammalian central nervous system and peripheral organs. The major ligand of the CB1-receptor is AEA – anandamide. This system is involved in many processes of the mammalian body, such as pain, sensation, mood, appetite, fertility or pregnancy. In this study, we analysed CB1-R and CB2-R levels in various parts of the CNS and female reproductive system.

**Methods:** The first stage of the study was organ extraction from lab mice. Subsequently, each sample was homogenized and centrifuged. We measured the total protein concentration and performed Western Blot assays on the extracts. Finally, the receptors were visualized using chemiluminescence. A detailed comparison of the results in tested tissues was performed.

**Results:** The study focused on two types samples of CNS tissue and two types of female reproductive tissue. A higher concentrations of the receptors were noted in the encephalon compared to the cerebellum and in uterine tissue compared to the oviduct. Receptors concentrations in the brain and uterus were similar.

**Discussion:** We confirmed a variable concentration of CB-1 receptor and CB-2 receptor in murine tissue. The levels of expression of these receptors in various parts of both systems are reflected in their function.

## A33: The antimicrobial potential of RNA G-quadruplexes

### Alison Hunt^1^, Catherine Greene^2^, Niall Stevens^2^

#### ^1^School of Medicine, Royal College of Surgeons in Ireland, Dublin, Ireland; ^2^RCSI Beaumont Hospital, Department of Clinical Microbiology, Dublin, Ireland

##### **Correspondence:** Alison Hunt

**Introduction:** RNA G-quadruplexes (RG4s) are stable structures that are unfolded in mammalian cells. In bacteria, they remain folded. Hence bacteria have undergone evolutionary depletion of endogenous RG4s possibly because they impair bacterial growth. The aim was to compare the potential antimicrobial activity of two RG4s against E.coli and S. aureus versus gentamicin and oxacillin.

**Methods:** Duplicate suspensions of E.coli (ATCC 25922) and/or S.aureus (ATCC 2160), set to a 0.5 McFarland Standard, were treated at 37oC overnight ±30% ethanol (positive control), 0.39-50μg/mL gentamicin or oxacillin (n=3) or 1.95-250μg/mL RG4s (n=1), specifically G3U (5’-UUU-GGG-UGG-GUG-GGU-GGG-3’), G3A2 (5’-UUU-GGG-AAG-GGA-AGG-GAA-GGG-UUU-3’) or a control RNA (5’-UUU-GAG-UGA-GUG-AGU-GAG-UGA-GUU-UU-3’) that does not form a G-quadruplex. The Miles and Misra method was used to determine the minimal inhibitory concentration (MIC) by calculating the number of colony-forming units per mL (CFU/mL). Competent E.coli cells (n=3) were prepared to increase their ability to uptake RG4s. These were transformed with 250μg/mL RG4s and CFU/mL were calculated.

**Results:** The MIC for (i) gentamicin against E.coli was 0.78-0.39μg/mL, (ii) oxacillin against S.aureus was inconclusive and (iii) no MIC could be calculated for the RG4s against E. coli. Compared to cells grown in TSB (1.89×109±4.09×108 CFU/mL), Ethanol killed 100% of the cells.

**Discussion:** There was no significant difference between the CFU/mL for G3U and G3A2 compared to the control RNA or negative control. This may be due to the RG4s not penetrating into the bacteria despite using competent cells, bacteriostatic rather than bactericidal activity of the RG4s, or that the RG4s are labile at 37oC in E.coli.

## A34: Influence of pretreatment with vitamins D and C on NAPDH-oxidase P22 subunit expression in hippocampus in animal model of stroke

### Ana Petronijevic^1^, Nela Puskas^2^, Vanja Radisic^3^

#### ^1^Histology and Embryology, University of Belgrade, School of Medicine, Institute of Histology and Embryology "Aleksandar Dj. Kostic", Belgrade, Serbia; ^2^University of Belgrade, School of Medicine, Institute of Histology and Embryology "Aleksandar Dj. Kostic", Histology and Embryology, Belgrade, Serbia; ^3^University of Belgrade, School of Medicine, Institute of Histology and Embryology, Histology and Embryology, Belgrade, Serbia

##### **Correspondence:** Ana Petronijevic

**Introduction:** Introduction: Occlusion of both common carotid arteries (global cerebral ischemia), followed by reperfusion, represents an animal model of stroke. NADPH oxidase (NOX) enzymes family physiologically produce reactive oxygen species. P22 is a membrane subunit present in NOX2 and NOX4 isoforms, that are strongly implicated in stroke. Hippocampus, a part of allocortex, is very sensitive to ischemia. Vitamin D has proven neuroprotective effects, while vitamin C (dehydroascorbic acid-DHA) has antioxidant activity. The aim of this experiment was to determine the effects of vitamin D and/or vitamin C on the expression of p22 subunit in different regions of Mongolian gerbil hippocampus.

**Methods:** Methods: Animals were divided into five groups: control group; I/R (10min ischemia/24h reperfusion), I/R+vitD3 (vitamin D during 7 days before I/R); I/R+DHA (vitamin C 30 minutes before I/R) and I/R+vitD3+DHA group. Brain sections from each group were stained on microscope slides with anti-p22 antibody. Density of p22 immunopositive neurites was determined using a B36 net. The results were analysed using the one-way ANOVA with the Tukey post-hoc test.

**Results:** Results: This experiment has shown that I/R leads to significant decrease in p22 expression in dentate gyrus compared to control group (p<0,05). I/R+vitD3 and I/R+vitD3+DHA groups had an increased expression of p22 in dentate gyrus compared to I/R group (p<0,01).

**Discussion:** Discussion: Vitamin D alone or with vitamin C, has shown protective potential and reversed changes of p22 subunit expression induced by I/R. Only p22 immunopositive neurites were counted, while p22 from glia and other brain cells were not included in this research.

## A35: The acceptability and utility of different diagnostic methods and sample types for the surveillance of trachoma in the Bijagos Islands, Guinea Bissau

### Ramandeep Sahota^1^, Emma Harding-Esch^2^

#### ^1^School of Medicine, King's College London, London, United Kingdom; ^2^London School of Hygiene and Tropical Medicine, Department of Infectious Diseases, London, United Kingdom

##### **Correspondence:** Ramandeep Sahota

**Introduction:** Trachoma is the leading infectious cause of blindness worldwide and is hyperendemic in the Bijagos Islands, a remote archipelago of islands that lie off the coast of Guinea Bissau. Once elimination of trachoma has been achieved in the Bijagos Islands it is imperative that a successful surveillance programme is in place. The aim of this study is to determine the acceptability and utility of different diagnostic tests and sample types that could be used for trachoma surveillance.

**Methods:** Semi-structured interviews of community members and stakeholders were followed by focus group discussions. These qualitative methods explored views on: experiences with trachoma, examining the eye for clinical signs, taking a conjunctival sample with a cotton bud, taking a blood sample, laboratory testing, health preferences within the community, and the challenges that may be faced by surveillance programmes.

**Results:** Community members expressed dissatisfaction with their health care experiences in relation to trachoma and in some cases were keen for different procedures that would be more acceptable and useful. In general, community members and stakeholders indicated a preference for the collection of samples which can be tested in the laboratory to detect trachoma infection. Despite this, stakeholders articulated their contentment with best current practice with a trend amongst community members to ultimately be happy with whichever intervention would give them good health.

**Discussion:** In this setting, diagnostic tests and sample types used for trachoma surveillance are accepted by communities to a degree. Appropriate sensitisation of communities prior to the implementation of a trachoma programme is crucial.

## A36: Influence of roll compaction/dry granulation on release characteristics from tablets containing an absorption modifying excipient

### Brian Dela Musoke^1^, Sam Maher^2^, Wessal Esharif^2^

#### ^1^School of Pharmacy, Royal College of Surgeons in Ireland, Dublin, Ireland; ^2^Royal College of Surgeons in Ireland, School of Pharmacy, Dublin, Ireland

##### **Correspondence:** Brian Dela Musoke

**Introduction:** The desire to develop medicines with good efficacy, safety and tolerability has come at the cost of greater structural complexity – which directly impacts aqueous solubility and intestinal permeability. Absorption modifying excipients (AMEs) are among the most widely tested approaches to improve oral bioavailability of drugs with low permeability. Co-presentation of AME and active at the intestinal epithelium improves bodily absorption through the creation of a diffusion gradient, and by enabling the AME to reach a threshold concentration for flux enhancement. However, leading AMEs in development can exhibit sub-optimal dissolution, which impedes optimal presentation.

**Methods:** This study assessed the effect of roll compaction/dry granulation on disintegration and dissolution of tablets containing two prototype actives (insulin or sodium fluorescein) and the novel AME, sodium undecylenate (C11:1). Granulates were prepared at three roll pressures (0.5, 1, and 3MPa) and two mesh sizes (8 and 12).

**Results:** Roll compaction/dry granulation had a major effect on flowability and compressibility of mixtures. A roll force of 1MPa with a mesh size of 8 particularly, achieved a 12 (Good) on the Carr Index and the Compressibility index, compared to 37 (Very Poor) by the admixed formulation. There was no difference between dissolution, disintegration or dose uniformity between tablets made using the admixed powder versus processed granulates. Inclusion of a super-disintegrant in the formulation helped to create an immediate release dosage, that created favourable conditions for optimal permeation enhancement action.

**Discussion:** This study shows that roll compaction/granulation parameters can be optimised to improve powder flowability and compressibility.

## A37: Recombinant expression and analysis of plasmodium falciparum erythrocyte membrane protein 1 (PfeMP1) protein domains that impact protein C pathways

### Giovanni Andrei Saw Ye Jeune^1^, Roger Preston^2^, Orla Fox^2^

#### ^1^Irish Centre for Vascular Biology, Department of Molecular and Cellular Therapeutics, Royal College of Surgeons in Ireland, Dublin, Ireland; ^2^Royal College of Surgeons in Ireland, Irish Centre for Vascular Biology, Department of Molecular and Cellular Therapeutics, Dublin, Ireland

##### **Correspondence:** Giovanni Andrei Saw Ye Jeune

**Introduction:** In recent studies, the protein C (PC) pathway was shown to be defective in Plasmodium falciparum-infected cerebral malaria patients. It is characterised by infected erythrocytes binding to the endothelial cell protein C receptor (EPCR) within the cerebral vasculature. Moreover, P. falciparum erythrocyte membrane protein 1 (PfEMP1), was shown to bind to EPCR with 200-fold higher affinity than that described for APC (KD ~0.3nM). PfEMP1 variants containing cysteine-rich inter-domain region, CIDRα1.1 and 1.4-1.8 domains, bind EPCR at a similar site to PC/APC, but with higher affinity. This study aims to generate CIDRα1 domains for functional analysis of their impact on PC/APC binding and cell signalling. Ultimately, this study will enable the generation of tools to regulate this interaction and inhibit vascular pathogenesis caused by P. falciparum-infected individuals with cerebral malaria.

**Methods:** This will be achieved by cloning a synthetic gene fragment encoding a His-tagged CIDRα1.4 domain into an E. coli protein expression plasmid to be used for recombinant expression, purification and molecular analysis.

**Results:** The CIDRα1.4 protein was generated and successfully expressed in the transformed E. coli cells. This was confirmed using Western blot analysis, in which an anti-His HRP-conjugated antibody detected CIDRα1.4 at the anticipated molecular weight of 40kDa. Complete purification of the CIDRα1.4 protein proved challenging despite altering the concentration of Imidazole in the wash and equilibration buffer.

**Discussion:** We were successful in expressing a synthetic gene fragment encoding the CIDRα1.4 domain. The complete purification of this recombinant protein requires further optimization prior to molecular analysis with EPCR.

## A38: Investigating the therapeutic potential of BET inhibitor JQ1 in models of invasive ductal carcinoma (IDC) and lobular carcinoma (ILC)

### Abdullah Almansouri^1^, Darran O'Connor^2^, Brian Mooney^2^

#### ^1^Medicine, University, Dublin, Ireland; ^2^University, Molecular and Cellular Therapeutics, Dublin, Ireland

##### **Correspondence:** Abdullah Almansouri

**Introduction**: Studies have shown that BET inhibitors have antitumor activity against various solid tumors, including tamoxifen resistant breast tumors. This study aimed on investigating the therapeutic effects for single and combined therapy of BET inhibitor JQ1 and tamoxifen on both IDC and ILC cell lines.

**Methods**: Following molecular characterization, the MCF7 and CAMA1 models were selected from a panel of breast cancer cell lines. For single-agent treatments, cells were treated with the established IC50 of each inhibitor for 72 hours, followed by the MTT assay to establish cellular viability. For combination treatments, cells were treated with varying doses of each inhibitor, and the MTT assay was again employed to establish cellular viability. Western blotting was used to examine PARP cleavage as an indication of JQ1 activity and progesterone receptor to examine tamoxifen activity.

**Results**: For single therapy, MCF7 showed moderate response to JQ1, but CAMA1 cells were more resistant. For the dual-agent “synergy” assay, synergy between JQ1 and tamoxifen was demonstrated in the MCF7 cells at high doses. Modest synergy was demonstrated in the CAMA1 cells. PARP cleavage following JQ1 treatment, as well as the combination treatment, was demonstrated in the MCF7 cell line. Furthermore, there was down regulation of progesterone receptors with tamoxifen and JQ1 for both cell lines.

**Discussion**: This study showed that MCF7 are more sensitive to BET inhibitor JQ1 compared with CAMA1. Although it was a small study, the results were promising. For future work, we aim on increasing our sample size and use JQ1 on tamoxifen-resistant cells.

## A39: Smokeless tobacco (snus) associated with periodontal diseases and oral malignity in Latvia

### Sintija Miļuna^1^, Juta Kroiča^2^, Māra Pilmane^2^, Aigars Reinis^2^, Dagnija Rostoka^2^

#### ^1^Faculty of Dentistry, Riga Stradiņš University, Riga, Latvia; ^2^Riga Stradiņš University, Department of Microbiology and Biology, Riga, Latvia

##### **Correspondence:** Sintija Miļuna

**Introduction:** Snus users are not fully informed about the possible harmful effects on the health from its use due to lack of information in Latvia. This paper examines whether there is any relevance between smokeless tobacco intake, periodontal diseases and oral malignity.

**Methods:** Using a questionnaire about smokeless tobacco consumption a heavy snus group, a light snus group and a control group were made. Oral biopsy samples were collected from heavy and light group snus users, and immunohistochemically tested for Ki67, PGP 9.5, CAB, IL-1, IL- 10. Periodontal pocket biofilms were collected from all groups and tested with RT-PCR. They were further analysed in order to determine the presence of pathogenic periodontal bacteria A. actinomycetemcomitans, P. gingivalis, T. forsythia, T. denticola and P. intermedia. The quantity of bacteria was expressed by the reference interval Lg/equivalents/sample. Statistical analyse made by the Mann-Whytney U test.

**Results:** Biopsy results showed parakeratosis, epithelial disorganization, vacuolization with prevalence of keratotic seborrhea, oral ulcerations. PGP 9.5 marked epitheliocytes, nerve fibres, fibroblasts were detected. Ki67 basal epitheliocytes were viewed. IL-10 was possessed in the epithelium. Il-1, CAB were absent. Microbiological examination showed presence of periodontal pathogens in heavy and light snus users group (p<0.05) but in the control group presence of periodontal pathogens were statically not significant (p>0.05).

**Discussion:** Smokeless tobacco spurs the persistent proliferation potential in the epithelium. Smokeless tobacco intake is related to periodontal diseases and can be associated with oral malignity. This study could build awareness in young adults about smokeless tobacco’s harmful effects.

## A40: Characterisation of CPEB-1 in relation to epilepsy

### Sarita Ankatiah^1^, Tobias Engel^2^, Laura de Diego-García^2^

#### ^1^Physiology and Medical Physics, Royal College of Surgeons Ireland, Dublin 2, Ireland; ^2^Royal College of Surgeons Ireland, Physiology and Medical Physics, Dublin 2, Ireland

##### **Correspondence:** Sarita Ankatiah

**Introduction:** Epileptogenesis is the process by which the normal brain undergoes changes, resulting in spontaneous recurrent seizures or epilepsy. Cytoplasmic polyadenylation element binding proteins (CPEBs 1-4) regulate cytoplasmic polyadenylation and translation through poly(A) tail elongation or repression and have been implicated in synaptic plasticity, long-term potentiation, learning and memory, processes affected during epileptogenesis. Since the association between cytoplasmic mRNA polyadenylation and epilepsy has yet to be investigated, this project aims to determine its role in status epilepticus (SE) and epilepsy.

**Methods:** Status epilepticus and chronic epilepsy were induced via intra-amygdala kainic acid injection and the mice were sacrificed after 1 hour, 24 hours and 14 days. Hippocampal CPEB1 expression was quantified using western blot, and sagittal hippocampal slices were examined by immunohistofluorescence to determine CPEB1 location.

**Results:** CPEB1 in the hippocampus demonstrated an increased expression after inducing SE. Even though the biggest increase in expression was observed after 4 hours and 24 hours, these results are not statistically significant. In the dentate gyrus of the hippocampus, CPEB1 was mainly expressed in microglia after 1 hour, 24 hours and 14 days post-SE, but not in neurons or astrocytes. Interestingly, in the cortex, CPEB1 was expressed in both microglia and neurons, with the most significant expression in neurons 1 hour and 24 hours after SE.

**Discussion:** An association exists between cytoplasmic mRNA polyadenylation and epilepsy, which requires further investigation to determine the nature of the interaction. The presence of CPEB1 in microglia may play a role in regulating gene expression related to neuroinflammatory processes during seizures.

## A41: Radiotherapy in the elderly; do they receive and tolerate the same treatment as younger patients with cancer?

### Claire Conlon^1^, Lorna Keenan^2^, Serena Brophy^2^, Mary Dunne^2^, Dr Orla McArdle^2^

#### ^1^Medicine, RCSI, Dublin, Ireland; ^2^St. Luke's Radiation Oncology Network, Medical, Dublin, Ireland

##### **Correspondence:** Claire Conlon

**Introduction:** The National Cancer Strategy for Ireland has identified that older patients with cancer are less likely to have tumour directed treatment versus younger patients. In order to evaluate radiotherapy provision, we detail the pattern of use of radiation oncology services by patients over 70 years of age and compare this to patients under 70 years of age.

**Methods:** This study includes patients aged >70, with a cancer diagnosis within the central nervous system, head and neck, lung, gastrointestinal and gynaecological categories. All patients seen for initial consultation in 2015 in our national cancer centre were included. Data on treatment intent and outcomes were prospectively collected via an automated database. A subset of patients aged <70 were analysed for comparison.

**Results:** 501 patients over 70 years of age were seen for radiotherapy opinion. The majority (85%) were treated, 67% with radical intent and 33% with palliative intent. Of the total population seen, <5% were not recommended for treatment because they were deemed unfit. Breaks in treatment due to toxicity were observed in <5% and treatment was discontinued in 2%. Patients under 70 (n=104) had similar overall treatment patterns and completion rates.

**Discussion:** The majority of older patients who were referred for consideration of radiotherapy were treated and completed treatment in a similar manner to younger patients. This demonstrates the successful treatment of older patients without exerting a burden of delayed or incomplete treatment and provides useful evidence to support the provision of tumour directed treatment for older patients.

## A42:Single burr hole in cranium as the final treatment for the compressive chronic subdural haematomas – time for “minimal invasive” steps in neurotrauma

### Muhamed El-Amin Zeid^1^, Adi Ahmetspahić^2^, Zulejha Omerbašić^3^, Salko Zahirović

#### ^1^Medicine- Neurosurgery, SSST and Clinical Center of University in Sarajevo, Sarajevo, Bosnia and Herzegovina; ^2^Clinical Center of University in Sarajevo, Neurosurgery, Sarajevo, Bosnia and Herzegovina; ^3^SSST and Clinical Center of University in Sarajevo, Medicine - Neurosurgery, Sarajevo, Bosnia and Herzegovina

##### **Correspondence:** Muhamed El-Amin Zeid

**Introduction:** Chronic subdural haematomas (CSDH) are one of the most common pathologies caused by head trauma, which leads to subdural bleeding with hematoma formation and symptoms, such as, headaches, neurological symptoms, or coma. For a long time the double Burr holes (DBH) procedure was and is the “gold standard treatment” for CSDH, however our prospective study focuses on the single Burr hole (SBH) procedure and its benefits. Therefore, does the single Burr hole procedure compared to the double Burr hole procedure provide better outcomes for both the patient and the surgeon?

**Methods:** 10 patients were treated for CSDHs by the (SBH) procedure with one hole and incision in the region of the parietal tuber. Patients presented with raised (ICP) and varying degrees of hemiparesis. The inclusion criteria only required the absence of multiple septal barriers in subdural space, identified by CT. A drain was left in the subgaleatic space for 48 hours post operatively.

**Results:** The postoperative period was uneventful with neurological symptom regression and no seizures. When discharged on the third postoperative day patients were instructed to consume antiepileptics for 7-14 days, perform follow up CTs at intervals of 7 then 30 days post operatively. The (SBH) approach showed the same clinical and radiologic results in the selected group of patients as in patients who underwent the (DBH) procedure.

**Discussion:** (SBH) should be option one for patients without septal barriers and patients undergoing anticoagulation therapy, due to; a shorter surgery, one cranial opening, same clinical and radiological results and a better cosmetic outcome.

## A43: The need for emergency care education: High school students’ exposure to health emergencies affecting confidence in emergency situations

### E Chin Mak^1^ , Henry A Curtis^2^

#### ^1^School of Medicine, RCSI, Dublin, Ireland; ^2^ Department of Emergency Medicine, Stanford Hospital, Palo Alto, CA, USA

##### **Correspondence:** E Chin Mak

**Introduction:** Laypersons are often bystanders of medical emergencies and are crucial to initiating immediate first aid. Education and training in emergency care therefore encourage laypersons to provide more efficient and fluent first aid at emergencies. Emergency care curriculums are often an optional course where students can choose not to participate in. However, training encourages fluidity of responding to health crises, allowing students to overcome inhibitors of providing help, like unfamiliarity, feeling scared and presence of other spectators, leading to higher helping rates . The study questions whether past experience in medical emergencies affect the student’s perceived comfort levels in providing immediate care. The interest in first-aid education amongst high school students is also explored.

**Methods:** High school students completed a survey for prior medical emergency experiences, comfort levels in assisting with multiple emergent situations and willingness to respond. Perception of the value of future first aid training was collected using a 10-point scale. Chi square tests were used for data analysis.

**Results:** Chi square tests showed association between past experience and comfort in assisting with medical care (p<0.001). The importance of school first aid training scored an average of 8.0 and 9.2, amongst the students with and without experience respectively.

**Discussion:** Those with prior experience in medical emergencies generally report higher comfort levels in assisting with urgent medical care, suggesting value in first aid training. The implementation of such training in high school curriculums provides exposure from an early age, aiding to overcome inhibitors and raise helping rates.

## A44: Old vs. new - The accuracy of targeted-SGA fetal weight estimation – results from the multicentre PORTO Study

### EmerRose Kealy^1^, Patrick Dicker^2^

#### ^1^Obstetrics & Gynaecology (OBGYN), Royal College of Surgeons Ireland, Dublin, Ireland; ^2^Royal College of Surgeons Ireland, Obstetrics & Gynaecology, Dublin, Ireland

##### **Correspondence:** EmerRose Kealy

**Introduction:** To determine if there is increased accuracy in switching to a targeted-SGA /LBW formula for fetal weight estimation in an SGA cohort.

**Methods:** This is a secondary analysis of The Prospective Observational Trial to Optimize Paediatric Health. This study recruited 1,200 singleton pregnancies with EFW <10th centile. Pregnancies underwent serial sonographic assessment of fetal weight and multi-vessel Doppler studies. Eight formulae developed specifically for SGA fetuses or low birthweight (LBW) infants were compared, in addition to the Hadlock III & IV (1985) and Intergrowth-21st (2017) formulae. The accuracy of formulae was assessed using percentage error and a prediction model was used to account for growth during the interval to delivery.

**Results:** Complete fetal biometry within one week of delivery was available in 588 pregnancies. The mean birthweight was 2,311g (SD=647g) and the median GA at delivery was 37.7 weeks (range=26.4-42.6 weeks). The Hadlock III, IV and Sabbagha III formulae all had almost no systematic bias. All three formulae had similar random errors (Interquartile range: 10-13%) and had the highest proportion of EFW within 10% of birthweight (77%). Percentage error was dependent upon both GA at scan (p<0.001) and time interval to delivery (p<0.001). The use of projection to delivery methods improved the accuracy of the other formulae but did not change the conclusions.

**Discussion:** Our study, which recruited pregnancies with EFW < 10th centile (Hadlock IV, 1985) suggests that there is no benefit to subsequently switching to formulae targeted for the SGA fetus or LBW infant.

## A45: Hadlock vs. Skupski: Estimating gestational age in twin pregnancies using NICHD standard (Skupski 2017)- Results of the multicentre ESPRiT twin study

### Fátimah Alaya^1^, Patrick Dicker^2^

#### ^1^Obstetrics & Gynaecology (OBGYN), RCSI Dublin, Dublin, Ireland; ^2^RCSI, Obstetrics & Gynaecology (OBGYN), Dublin, Ireland

##### **Correspondence:** Fátimah Alaya

**Introduction:** To assess the performance of the new NICHD formula for gestational age (GA) determination in a twin population.

**Methods:** This is a secondary analysis of the ESPRiT Study. Two subgroups of the ESPRiT cohort were used for GA determination, and had full biometry assessments in the 2nd and 3rd trimesters. (1) IVF/ICSI-conceived pregnancies (120) (2) Non-IVF pregnancies with Crown-Rump Length (CRL) assessed in the first trimester (276) Comparisons were made between IVF embryo-transfer-date (ETD) versus CRL-dating, ETD and later pregnancy biometry-dating and CRL, via Hadlock (1992) and later pregnancy biometry dating via Hadlock (1984) and NICHD/Skupski (2017). GA accuracy estimation was via the mean (systematic error) & SD (random error) of differences in GA.

**Results:** The NICHD formula systematically underestimates GA in twin pregnancies by 1-2 weeks in the 2nd and 3rd trimesters in IVF and spontaneously conceived twin pregnancies, resulting in more spontaneously conceived pregnancies being re-classified as sub-optimally dated. In the IVF population, 1st trimester CRL dating was more accurate in the smaller (CRL) twin, overestimating gestational age by 2-3 days on average. Both smaller and larger twins performed well using later pregnancy biometry dating (Hadlock) in the 2nd & 3rd trimesters, with the average twin gestation having minimum errors. In the spontaneous conception population with CRL assessments, the larger (EFW) twin performed the best in the 2nd and 3rd trimesters using fetal biometry.

**Discussion:** The NICHD method for gestational age determination cannot be recommended as an alternative to the Hadlock (1984) method in the 2nd/3rd trimester for twin pregnancies.

## A46: Does pharmacoinvasive strategy effectively bridge the gap in the absence of a catheterisation lab in STEMI patients?

### Sean Maher^1^, Liviu Ghilencea^2^, Serban Balanescu^2^, Doina Dimulescu^2^, Andreea Popescu^2^

#### ^1^Cardiology, Carol Davila University of Medicine and Pharmacy, Bucharest, Romania; ^2^Carol Davila University of Medicine and Pharmacy/Elias Emergency University Hospital, Cardiology, Bucharest, Romania

##### **Correspondence:** Sean Maher

**Introduction:** ST-segment Elevation Myocardial infarction (STEMI) is one of the leading causes of death worldwide. International guidelines clearly state that Primary Percutaneous Coronary Intervention (PPCI) is the gold standard of treatment. However, due to a lack of cardiac catheterisation services, many patients receive thrombolytic therapy at a non-interventional hospital before being transferred to a PCI capable hospital. This single centre observational study investigated the efficacy and safety of Pharmacoinvasive Strategy (PIS) versus PPCI in the context of the Romanian National STEMI program.

**Methods:** From January 1st – May 31st 2018 forty-seven consecutive patients from surrounding county hospitals were enrolled in this study and divided into two groups; The first group underwent thrombolysis in the first contact hospital before being referred to our PCI center (PIS group) while the second group was transferred directly for PCI (PPCI group).

**Results:** Cardiovascular risk factors were present in the vast majority of patients with no major differences between the two groups. Left ventricular systolic dysfunction (LVEF less than 40%) was more frequent in the PPCI group. The number of diseased vessels including the culprit lesion was similar in both groups. In-hospital deaths, length of hospital stay and in hospital MACE were higher in the PPCI group, than in the PIS group.

**Discussion:** PIS had better outcomes than PPCI when long distances to catheterization labs exist. The results suggest that thrombolysis should be performed in non-PCI centers with referral to a 24/7 PCI capable hospital as soon as possible. PIS is a viable alternative that saves lives.

## A47: Are ER+ breast cancer cells de novo resistant or do they acquire their resistance to Tamoxifen therapy?

### Mansi Shah^1^, Leonie Young^2^

#### ^1^Royal College of Surgeons in Ireland, Dublin, Ireland; ^2^Royal College of Surgeons in Ireland, Endocrine Oncology Research Group, Ireland

##### **Correspondence:** Mansi Shah

**Introduction:** Breast cancer is one of the leading causes of death in women. Anti-endocrine therapies such as Tamoxifen are often used to treat it. However, 30% of them develop resistance, posing a significant challenge clinically. Early events in endocrine resistance are poorly understood. Previous work in this lab has identified SSEA1+ cells as drivers of tamoxifen resistance in ER+ breast cancer. It was found that these cells were not de novo resistant, but rather acquired this feature. This led us to asking ‘’Are ER+ breast cancer cells in additional tissue culture models and patient tissue samples de novo resistant or do they acquire their resistance to Tamoxifen therapy?”

**Methods:** Tissue culture and flow cytometry was performed on the T47D cell line in vehicle, estrogen and estrogen and Tamoxifen. The cells were subsequently stained with DCO and anti-SSEA1-APC to measure progress through the cell cycle with respect to SSEA1 marked sub-populations. Immunohistochemistry/Immunofluorescence was also performed using primary and secondary antibodies against SSEA1 and Ki-67.

**Results:** Analysis of the T47D cell line confirmed the previous findings that SSEA1+ cells are not de novo resistant. Unfortunately, there was insufficient proliferation in ex vivo tumour samples to confirm or reject the hypothesis.

**Discussion:** This study has helped in understanding this particular cell type (SSEA1+)that drives endocrine resistance in breast cancer. RNA sequencing could further be employed to understand key genes that differ between SSEA1+ and SSEA- cell types which could potentially be used as biomarkers of prognosis or targets of therapy in the future.

## A48: Prevalence of Diabetes Mellitus secondary to pancreatic diseases (type3cDM): A 13-year retrospective cohort study in a university hospital

### Kameron Chatoor^1^, Qurat Ul Ain^2^, Sinead Duggan^2^, Kevin Conlon^2^

#### ^1^Medicine, Royal College of Surgeons in Ireland, Dublin, Ireland; ^2^Trinity College Dublin, Tallaght University Hospital, Department of Surgery, Tallaght, Dublin, Ireland

##### **Correspondence:** Kameron Chatoor

**Introduction:** The American Diabetes Association (ADA) classified Diabetes Mellitus (DM) secondary to pancreatic diseases as Type3cDM. Typically, Type3cDM occurs in patients with chronic/acute pancreatitis, pancreatic cancer, and pancreatic surgery. However, due to poor awareness, Type3cDM patients are frequently misclassified as type 1/2 DM, and may therefore be mismanaged. We aimed to cross-match a large DM database with a pancreatic disease database to determine how many patients had both pancreatic disease and DM, and to identify how many patients had a diagnosis of DM following pancreatic disease, suggestive of type3cDM.

**Methods:** This was a retrospective observational study. For a 13-year period (2004-2017) the Department of Surgery Pancreatic Database (PD) and Diamond Diabetes Database (DDD) in Tallaght University Hospital were cross-matched for common patients.

**Results:** The DDD had 12,000 patients for 2004-2017, while the PD had 2,048 patients. Cross-matching showed that there were 287 common patients with both pancreatic disease and DM. Of these, 194 patients (67.6%) were diagnosed with DM after pancreatic disease diagnosis, suggestive of true type3cDM. However, only 56 patients (19.5%) had been classified as type3cDM. The most common aetiology was acute pancreatitis (45%). For the remaining 231 patients, 192 (66.9%) were Type 2 and 39 (23.5%) Type 1.

**Discussion:** While more than half of patients were thought to have Type3cDM they had not been classified as such, despite ADA guidelines. Type3cDM is difficult to manage, particularly when coupled with the other complications of pancreatic diseases, such as malabsorption. Correct classification would allow optimal management, and improved disease understanding.

## A49: Clinical audit on IV cannula use at Beaumont Hospital

### Parijot Kumar^1^, Arnold Hill^2^

#### ^1^Surgery, Royal College of Surgeons in Ireland, Dublin, Ireland; ^2^Royal College of Surgeons in Ireland, Surgery, Dublin, Ireland

##### **Correspondence:** Parijot Kumar

**Introduction:** Peripherally Inserted Venous Catheters (PIVC) are most often used to facilitate the administration of intravenous (IV) medications or fluids. We present a prospective clinical audit on IV cannula use from 3 surgical wards, AB Clery, Phoenix and Finbar’s Day Ward, at Beaumont hospital, in order to assess adherence to local hospital guidelines, from which our standards were derived.

**Methods:** We collected data from 100 patients over a period of 6 weeks, from 8th January 2018 to 16th February 2018. Handwritten raw data was transcribed onto a Microsoft Excel spreadsheet and processed into relevant graphs and figures.

**Results:** We found that 96% of our cohort had a documented indication for cannula insertion, 79% of cannulas were inserted into distal veins, 62% of cannulas had an insertion and removal date documented, 65% of cannulas were removed within 72 hours, 23.5% of cannulas sited in emergency situations were removed with 24 hours and 66.7% of cannulas with signs of phlebitis were removed in 24 hours.

**Discussion:** We have recommended IV cannula review on every ward round, allowing cannulas inserted in A&E to remain for 72 hours in the absence of signs of phlebitis, and requesting nurses to prompt a medical review of a cannula that has been in-situ for 72 hours. We have made a poster to better implement these recommendations. We further recommend performing a re-audit with the above changes to identify any quality improvement.

## A50: A novel ELISA to detect autoantibodies in systemic lupus erythematosus (SLE): using Secondary NEcrotic Cells (SNECs)

### Erica O'Sullivan^1^, Mona Biermann^2^, Louis Munoz^2^, Martin Herrmann^2^

#### ^1^School of Medicine, RCSI, Dublin, Ireland; ^2^University Hospital Erlangen, Immunology and Rheumatology, Medical Clinic 3, Erlangen, Germany

##### **Correspondence:** Erica O'Sullivan

**Introduction:** SLE, an inflammatory autoimmune disease involves auto-antibodies, immune-complex deposition and multi-organ damage. Diagnosis includes Anti-Nuclear-antibody testing (Sensitive, not specific for SLE), followed more specific autoantibody testing (double-stranded-DNA (dsDNA), Ro, La, U1-Ribonucleoprotein). A deficiency of apoptotic cell-clearance plays an important role in the pathogenesis of SLE, thus complement pathway deficiencies confer a higher risk. We aimed to develop a highly specific test, utilizing secondary-Necrotic-cell (SNEC)-derived material as a substrate, for the detection of SLE-autoantibodies (AAb) and compare SNECs derived from whole-blood to cultured-cells.

**Methods:** Ethical-approval' obtained. Serum collected from Normal/Healthy Donors (NHD), patients with SLE, other auto-immune-disorders (OAD). Peripheral-Blood-Mononuclear-cells (PBMCs) isolated from whole-blood. Jurkat-cells cultured. Apoptosis induced by UV-radiation. Anti-SNEC-AAb measured in serum of SLE (n=155), NHD (n=89), OAD (n=169) using SNEC-based indirect enzyme-linked immunosorbent assay (SNEC-ELISA). SNEC-ELISA compared to routine anti-dsDNA diagnostics: radioimmunoassay (RIA) and nucleosome-based-ELISA (NcX-ELISA). SNEC-derived from whole-blood (primary-SNEC) compared to cultured cells (jurkat-SNEC).

**Results:** Primary-SNEC-ELISA distinguished SLE with 98.9% specificity and 70.6% sensitivity from NHD; surpassing Jurkat-SNEC (98.25%, 43.37%), anti-dsDNA: RIA (97.6%, 37.9%)/ NcX-ELISA (98.4%, 43.1%). Positive Anti-SNEC-AAbs on SNEC-ELISAs discriminated SLE from rheumatoid arthritis, anti-phospholipid syndrome, spondyloarthropathy and systemic sclerosis.

**Discussion:** Primary-SNEC-ELISA detects SNEC-AAb in SLE-Serum with high sensitivity and specificity, surpassing current autoantibody testing in routine diagnostics. Stressing SNECs role in SLE-pathogenesis and potential for Diagnosis/Monitoring. A new cohort of NHD-Serum samples were compared between Jurkat-SNEC and Primary-SNEC. Additionally, Jurkat-Cells are Immortalised T-cells, thus distinct from Primary-PBMCs. This may explain the difference in assay sensitivity between primary-SNEC and Jurkat-SNEC. Future Research can optimise SNEC-ELISAs and further standardise testing.

## A51: An audit on IV cannula use on the surgical wards of Beaumont Hospital

### Hiba Makhdum^1^, Arnold Hill^2^

#### ^1^Department of Surgery, Royal College of Surgeons Ireland, Dublin, Ireland; ^2^Royal College of Surgeons Ireland, Department of Surgery, Dublin, Ireland

##### **Correspondence:** Hiba Makhdum

**Introduction:** Peripheral intravenous cannulation (PVC) can be associated with multiple complications. Various criteria are taken into account in the care of PVCs in order to minimise these complications. This study was conducted to evaluate the compliance rates and the significance of these criteria.

**Methods:** A standards-based clinical audit was conducted on the two surgical wards of Beaumont Hospital. Standards and criteria were set by the Beaumont Hospital policy on insertion and maintenance of PVCs. A cohort of 104 cannulas was collected over a 6-week period. Microsoft Excel was used to compile and analyse the data.

**Results:** 40% of cannulas inserted on the ward were removed within 72 hours. 3% of cannulas inserted in an emergency setting were removed within 24 hours. Early signs of phlebitis were present in 6% of cannulas changed within 72 hours, 9% of cannulas left in for longer than 72 hours and 3% of emergency setting cannulas left in for longer than 24 hours. Early stages of phlebitis were seen in 1% of the audited cannulas.

**Discussion:** 1. PVCs inserted on the ward may remain in-situ up to 96 hours, provided there are no clinical signs of phlebitis. 2. PVCs inserted in an emergency setting may remain in-situ up to 72 hours, provided there are no clinical signs of phlebitis. 3. A cannula should be checked a minimum of once daily and this should be documented. 4. Regular cannula care awareness teaching sessions should be conducted as well as yearly cannula care audits.

## A52: Drug utilization study in epilepsy in a tertiary care hospital

### Nethmie Chandrarathna^1^, Amrita Parida^2^, Uppor Adiga^2^, Arjun Sharma^2^

#### ^1^Pharmacology, Kasturba Medical College Mangalore, Manipal Academy of Higher Education, Mangalore, India; ^2^Kasturba Medical College Mangalore, Manipal Academy of Higher Education, Mangalore, India

##### **Correspondence:** Nethmie Chandrarathna

**Introduction:** Pharmacotherapy in epilepsy requires the drugs to be taken for a long time. These drugs have a negative impact on cognition, are potentially teratogenic and considered to be not so safe. Moreover, to remain seizure free, the patient often requires more than one drug. Additionally, these patients would also require drugs other than antiepileptics in presence of concomitant illnesses. Such polypharmacy comes with its own drawbacks like higher incidences of drug interactions, adverse effects and increased economic burden on the patient. In the current study, we analysed the drug utilization pattern in different types of epilepsy including the extent of polypharmacy

**Methods:** Prescriptions of 100 epileptic patients admitted in a tertiary care hospital were scrutinised. Descriptive statistics was used to analyse the data

**Results:** Sample consisted of 66 paediatric and 34 adult patients. The most frequent type of epilepsy encountered was generalised tonic-clonic seizures. Commonest antiepileptic drug (AED) prescribed was phenytoin (40%) and levetiracetam (38%) was the most frequent add-on drug. The average number of drugs prescribed to the patients was 6 which included drugs given for concomitant illnesses as well. Most of the patients (62%) required multiple antiepileptic drugs for seizure control

**Discussion:** It is evident from this study that epileptic patients are subjected to a large number of drugs which puts them at a risk of potential drug interactions and adds to the cost of treatment. Hence, rational prescription should be encouraged and the use of each drug should be justified.

## A53: Gene regulation following acute seizure and chronic epilepsy

### Razi Alalqam^1^, Tobias Engel^2^, Giorgia Conte^2^

#### ^1^Neurology, RCSI, Dublin, Ireland; ^2^RCSI, Neurology, Dublin, Ireland

##### **Correspondence:** Razi Alalqam

**Introduction:** Epilepsy is a disease characterized by abnormal neural activity in the brain which cause seizures. What gene changes underlie epilepsy development, however, are incompletely understood. Thus, the purpose of this study is to identify genes which change their expression during acute seizures (status epilepticus) and chronic epilepsy.

**Methods:** To identify these genes we injected kainic acid into the amygdala of mice a to induce status epilepticus. The hippocampi were collected at two time-points: 8 hour (8 h) after status epilepticus (acute phase) and at 14 day (14 d) after status epilepticus when all mice experience epileptic seizures. A control group was also present. Using microarray we were able to identify the upregulated and downregulated genes in both 8h and 14 d samples. Next, KEGG pathway analysis was performed to identify relevant cascades. Finally, RT-qPCR was carried to validate the most significant genes.

**Results:** Upon many upregulated and downregulated genes, our results show a significant downregulation of the ITPR1 gene in 14 d samples in relation to the control group. Furthermore, KEGG pathway analysis revealed the physiological role of ITPR1 in the calcium signaling pathway.

**Discussion:** Our data offers new insight towards abnormal gene expression in epilepsy. The downregulation of ITPR1 gene in epilepsy may serve as a negative feedback loop to counteract increased hyperexcitability.

## A54: PAD2 enhances airway degradation due to MMP-12 in emphysema

### Rashed Alshuhoumi^1^, Emer Reeves^2^

#### ^1^Respiratory, Beaumont Hospital, Dublin, Ireland; ^2^ RCSI- Beaumont Hospital, Respiratory, Dublin, Ireland

##### **Correspondence:** Rashed Alshuhoumi

**Introduction:** Alpha-1-antitrypsin (AAT) is a protein synthesized by the liver, and its deficiency could lead to emphysema. Emphysema can be caused by smoking or inherited through alpha-1-antitrypsin deficiency (AATD). The aim of this project is to examine the role of PAD2 in airway destruction, and to show how elastin degradation by MMP-12 is affected by PAD2.

**Methods:** MMP-12 was treated with PAD2 to determine whether PAD2 could influence the activity of MMP-12 in a method called FRET assay. Additionally, two reactions were set up between elastin and PAD2. One reaction contained calcium, which is required to activate PAD2. Hence, citrullinated elastin was produced from the reaction that contained calcium. Western blotting was used to detect citrullinated elastin and elastin itself.

**Results:** After treating MMP-12 with PAD2, the results showed that the activity of MMP-12 was not affected. Also, MMP-12 was added to tubes containing elastin to see the difference in degradation. We found that the citrullinated elastin was more resistant to degradation. The same experiment was repeated using PAD inhibitors and it showed that the citrullinated elastin is more susceptible to degradation.

**Discussion:** Degradation of elastin by MMP-12 was due to the citrullination of elastin. MMP-12 requires calcium to function. This could explain the increase in resistance of citrullinated elastin to MMP-12. Calcium, introduced with MMP-12, might have activated PAD2 in the reaction that had no calcium in it. Therefore, the same experiment was repeated with a PAD inhibitor in the reaction that previously lacked calcium to ensure that PAD2 does not get activated.

## A55: Interaction between nicotine and caffeine in memory acquisition

### Adelina Sorescu^1^, Isabel Ghita^2^, Tudor Enache^2^, Oana Coman^2^

#### ^1^Medicine, Carol Davila University of Medicine and Pharmacy, Bucharest, Romania; ^2^Carol Davila University of Medicine and Pharmacy, Department of Pharmacology and Pharmacotherapy, Bucharest, Romania

##### **Correspondence:** Adelina Sorescu

**Introduction:** Caffeine, as well as nicotine is known to influence the memory and learning processes. In this study, we aimed to determine their interaction and their effects on the acquisiton phase of memory (AQ).

**Methods:** 4 groups (n=12) of 250 g Wistar rats underwent a Y maze test. Pre-test caffeine, nicotine, their combination (in different doses) or saline solution were administered. The percentage of time spent in the unknown arm (PT) was measured. Surface EEGs were performed. The global power of the waves and their amplitude were determined by a Fourrier analysis. The t-Student test was performed in Microsoft Excel. The local ethical committee approved this study.

**Results:** 0.5 mg/kg, 10 mg/kg and 25 mg/kg of intraperitoneal caffeine, determined a 4%, a 3% and a 12% increase in AQ and increased PT (35.09%, 34.8%, 38,1%, respectively), compared to the control group (33.1%) (p>0.05). Administering 0.1 mg/kg, 0.2 mg/kg, 0.4 mg/kg of nicotine determined a 4%, a 3% and a 19% increase in AQ and prolonged PT (35.09%, 34.8% and 40.29%, respectively), compared to the control group (33.18%) (p<0.05). The combination of caffeine (25mg/kg) and nicotine (0.4 mg/kg) increased AQ by 10% and PT (38.86%) compared to the control group (p<0.05). Both substances modified the global power of the waves and their amplitude, differently on the posterior, anterior and lateral channels of the animal cortex.

**Discussion:** Both caffeine and nicotine exert a dose-related effect on AQ. Their combination enhanced memory processes as well, but no interaction was seen between the two substances.

## A56: Clinical and epidemiological peculiarities of Parkinson’s disease in West Ukraine

### Ivanna Yaremchuk^1^, Oksana Yaremchuk^2^

#### ^1^Department of Nervous Diseases, Psychiatry and Medical Psychology, Higher State Educational Institution “Bukovinian State Medical University”, Chernivtsi, Ukraine; ^2^Higher State Educational Institution “Bukovinian State Medical University”, Department of Nervous Diseases, Psychiatry and Medical Psychology, Chernivtsi, Ukraine

##### **Correspondence:** Ivanna Yaremchuk

**Introduction:** Parkinson’s disease (PD) is one of the most common neurologic disorders, affecting approximately 1% of individuals older than 60 years. The aim of this study was to investigate prevalence and peculiarities of clinical characteristics of PD in the Chernivtsi region of Ukraine among different gender and age groups.

**Methods:** This 5-year retrospective review involved the investigation of the Register of Neurological Diseases in Chernivtsi region between the years 2013-2017. We used epidemiological and statistical methods of research.

**Results:** According to the Register there were registered patients with: PD – 384 patients (42.3 per 100,000 population), secondary parkinsonism – 204 (22.5), neurodegenerative disease with parkinsonism syndrome – 26 (2.9). Among patients with PD was found 46.25% men and 53.75% – women. UPDRS index increase depends on age and stage of PD. Indicator of disruption of motor aspects of daily activity in older group was 2.4 times more than in young group. Our study showed direct correlation between age and severity of clinical manifestations. 16.2% of patients were treated by dopamine receptor antagonists, 21.8% of patients - by levodopa, 13.3% - by cholinolytics drugs, 10.5% - by amantadine, 38.2% by combination of two or more antiparkinson drugs.

**Discussion:** By means of our research PD is more frequent among women. The most frequent age is between 60 and 74 years old. The majority of patients in the first examination already had Hoehn and Yahr Stage 2.0. Probably this is the consequence of inadequate awareness about early peculiarities of parkinsonism among population.

## A57: Evaluation of the benefit of family screening in MZ alpha-1 antitrypsin deficiency

### Ming Hei Fu^1^, Tomás Carroll^2^, Noel McElvaney^2^

#### ^1^Alpha One Foundation Ireland, Beaumont Hospital, Dublin, Dublin; ^2^Beaumont Hospital, Alpha One Foundation Ireland, Dublin, Dublin

##### **Correspondence:** Ming Hei Fu

**Introduction:** Alpha-1 antitrypsin deficiency (AATD) is a genetic condition caused by mutations in the SERPINA1 gene coding for the alpha-1 antitrypsin protein. AATD is a known risk factor for COPD in adults and liver disease in children. Z is the most common deficient genetic mutation in Ireland and M is the normal version of the gene. MZ phenotype patients have a decreased level of protein compared to normal (MM) and they are shown have a more rapid lung decline than MM smokers. There are estimated to be 170,000 MZ patients in Ireland but it is underdiagnosed. The number of patients diagnosed through family screening is increasing and this study aims to evaluate its impact.

**Methods:** We used data collected from patients attending the National Centre of Expertise for Alpha-1 at Beaumont Hospital to build a profile in the National AATD Registry. Patients completed a questionnaire about their social and medical history, a complete spirometry and a radiological scan (CT thorax). Prism and MS excel were used for data analysis.

**Results:** Patients in the family screening group were shown to be younger with better lung function. A smaller proportion of them have respiratory problems and they have significantly lower pack years.

**Discussion:** Through family screening, AATD patients are diagnosed at a younger age therefore allowing for earlier intervention. COPD is the number one cause of admission to emergency departments each winter in Ireland. Therefore, diagnosing and better managing MZ patients’ respiratory problems can potentially ease the burden of chronic respiratory disease on the healthcare system.

## A58: Surgical intervention for infective endocarditis: Mechanical or biological prosthesis? A meta-analysis

### Alexander Reynolds^1^, Sanjay Asopa^2^, Nicola King^2^, Clinton Lloyd^2^

#### ^1^School Of Biomedical Science, Faculty Of Medicine and Dentistry, University Of Plymouth, Plymouth, United Kingdom; ^2^University Hospitals Plymouth, South West Cardiothoracic Centre, Plymouth, Devon

##### **Correspondence:** Alexander Reynolds

**Introduction:** Infective Endocarditis has a prevalence of 1 in 30,000 people per year. Controversy exists as to whether superiority exists between mechanical valves (MP) or biological valves (BP)- specifically xenografts. The aim of this meta-analysis was to identify surgical parameters and determine whether superiority exists between either mechanical or biological prosthesis.

**Methods:** PubMed, Science Direct and the University of Plymouth Primo Database were searched for suitable studies. Studies were compared to identify common parameters that could be used to statistically compare the efficacy of both valve types from different clinical perspectives. Data from these studies was analyzed using Revman 5.3.

**Results:** Six studies were accepted for participation following scrutiny from the authors based on an agreed selection criterion. Seven viable parameters (>2 studies) were identified and put to meta-analysis. Age was not stratified between MP and BP samples in any study. MP was found to have a significantly lower in-hospital mortality (P <0.0001), lower morbidity in terms of sternal wound infection (P = 0.05). However, less favorable in terms of postoperative thromboembolism (P = 0.04).

**Discussion:** MP is clinically superior to BP for in-hospital mortality favours MP and for incidence of thromboembolism. Limitation in sample size restricts the internal validity of this data. Further research is needed for replication and explanation of data.

## A59: Synthesis of novel thiazolidinedione derivatives with a dual pharmacophore

### Eoin Campion^1^, James Barlow^2^

#### ^1^Department of Chemistry, Royal College of Surgeons in Ireland, Dublin, Ireland; ^2^Royal College of Surgeons in Ireland, Department of Chemistry, Dublin, Ireland

##### **Correspondence:** Eoin Campion

**Introduction:** Depression and diabetes are both major medical conditions, which effect over 300 million and 422 million people worldwide respectively.. Diabetic patients are twice as likely to have comorbid depression when compared to non-diabetic patients. Selective serotonin reuptake inhibitors (SSRIs) are commonly used to treat depression. However, an adverse effect of long-term SSRI treatment is weight gain and increased appetite, particularly for carbohydrates. This can be complicate treatment of type 2 diabetes. The idea behind this synthesis, was to create an SSRI antidepressant with an additional thiazolidinedione pharmacophore. Thiazolidinedione is used to increase insulin sensitivity in type 2 diabetes. Therefore, the drug to be synthesized could potentially be used to specifically treat comorbid depression in type 2 diabetes, simultaneously increasing insulin sensitivity to better control of blood glucose levels in type 2 diabetes.

**Methods:** Retrosynthetic analysis was used to determine a suitable organic synthesis pathway to synthesize the target drug molecule. The synthesis began using a commercially available starting material. This was reacted in multiple steps, with use of a microwave synthesizer to give an SSRI analogue, which was characterized using NMR sprectroscopy. An oxidation of methylbenzene group was then attempted, which was necessary to attach the thiazolidinedione.

**Results:** The oxidation reaction failed, and instead lead to the destruction of the molecule.

**Discussion:** Due to the failed oxidation, It was concluded that this organic synthesis pathway was not worth further investigating for its intended purpose. An alternative synthesis pathway was then investigated to pursue the synthesis of it the thiazolidinedione dual pharmacophore drug.

## A60: An investigation of the main modifiable risk factors associated with breast cancer among supplement users and non-users

### Emma Parker^1^, Jamie Madden^2^

#### ^1^School of Medicine, Royal College of Surgeons, Dublin, Ireland; ^2^Department of Epidemiology, Royal College of Surgeons, Dublin, Ireland.

##### **Correspondence:** Emma Parker

**Introduction:** Vitamin D has recently been associated with lower breast cancer risk but the evidence is not clear. The aim was to examine modifiable breast cancer risk factors and to explore their distribution between vitamin D users and non-users in an Irish context.

**Methods:** Two separate cohorts were used in this study which included a healthy, general population based cohort and a breast cancer cohort. BREAST-PREDICT, conducted a prospective study which involved recruiting new breast cancer patients at diagnosis (n=715). For the healthy cohort, the Irish Longitudinal Study of Ageing (TILDA), was used, which is a large (n ≈ 8,000), ongoing, nationally representative study. Both cohorts had questionnaires which contained self-report information on vitamin D and supplement use. Chi-square tests and t-tests were used to compare differences between vitamin D/supplement users and non-users.

**Results:** In BREAST-PREDICT smoking was higher among vitamin D users compared with non-users (p=0.01). In both cohorts, overall supplement users were slightly older on average than non-users; BREAST-PREDICT (t=-8.88, p<0.01), TILDA (t=-2.7, p<0.01). There was a significant association between high exercise levels and supplement use (p<0.01) in TILDA. More supplement non-users were obese compared with supplement users in TILDA (p<0.01), the opposite was observed in BREAST-PREDICT (p=0.6).

**Discussion:** While a direct statistical comparison between the two groups could not be concluded, some statistically significant associations between modifiable factors and supplement use in both cancer patients and the general population were determined. Future studies should include pharmacy claims data to overcome self-report biases and vitamin D serum levels could be obtained.

## A61: Effect of long term storage on disintegration and dissolution of an overencapsulated tablet of penicillin V

### Joseph Saleh^1^, Sam Maher^2^, Abel Wakai^3^, John Hayden^2^

#### ^1^Pharmacy, Royal College of Surgeons in Ireland, Dublin, Ireland; ^2^Royal College of Surgeons in Ireland, Pharmaceutics, Dublin, Ireland; ^3^Beaumont Hospital, Dublin, Ireland

##### **Correspondence:** Joseph Saleh

**Introduction:** Over-encapsulation is a technique used to conceal the identity of a tablet product for blinding in randomised controlled clinical trials. It involves insertion of the tablet in to an opaque capsule shell along with a backfill excipient to prevent rattling. Clinical investigators are required to provide the regulator with evidence that overencapsulation does not alter stability for the duration of the product shelf life. This study assessed the effect of overencapsulation on long term storage and accelerated storage on release from the modified product.

**Methods:** Penicillin V tablets were overencapsulated in hard gelatin capsules (DNcaps® AAA, Capsugel, USA) with a set quantity of backfill (talc). Batches of unmodified tablets and overencapsulated tablets were stored for 0, 1, 2, 3 and 6 months at accelerated storage conditions (40 ± 2°C, relative humidity: 75 ± 5%) or 0 and 12 months at ambient storage conditions. Disintegration and dissolution testing were performed according to the European Pharmacopoeia.

**Results:** There was 60-90% release of penicillin V between 5-15 minutes from unmodified tablets that were stored for 0-to-2 months under accelerated storage conditions. There was a small lag in release between unmodified tablets and over-encapsulated tablets. There was complete release of penicillin V from the unmodified/modified tablets stored for 12 months. Interestingly, there was slower release of penicillin from the unmodified tablet compared to OET at 15 min.

**Discussion:** These findings suggest that Long term storage and accelerated storage conditions did not alter release characteristics of overencapsulated tablets compared with unmodified tablets.

## A62: Risk factors and complications associated with teenage pregnancy in the North Eastern part of Romania

### Ana Grigore^1^, Mihaela Grigore^2^, Diana Spînu^2^, Domnica Cretu^2^

#### ^1^General Medicine, University of Medicine and Pharmacy "Grigore T.Popa" Iasi, Iasi, Romania; ^2^University of Medicine and Pharmacy "Grigore T.Popa" Iasi, Gynecology and Obstetrics, Iasi, Romania

##### **Correspondence:** Ana Grigore

**Introduction:** Introduction: Teenage pregnancy has multiple consequences in terms of maternal health, child health and the overall well-being of society. The purpose of the paper is to evaluate sociocultural issues and other risk factors associated with teenage pregnancy.

**Methods:** Methods: We have conducted a retrospective study on a number of 526 pregnant teenagers (13-17 years old) hospitalized between 2015 and 2017 at the Cuza Voda Hospital in Iasi, Romania. We have analysed several sociocultural factors as well as ante-, intra- and post-partum complications.

**Results:** Results: Out of a total of 526 patients 96 (18,25%) are from the urban area and 430 (81,75%) are from the rural area. Only 120 (22,81%) patients were followed up by general practitioners or obstetricians. The main antenatal complications were: anemia 200 cases (38,02%), preterm labour 54 cases (10,26%), arterial hypertension 23 cases (4,37%), placenta praevia 5 cases (0,95%). In 162 cases (30,79%) a c-section was performed. The main intra-partum complications were: feto-pelvic disproportion 70 cases (13,30%), premature rupture of membrane 40 cases (7,6%), umbilical cord pathology 36 cases (6,84%), cervical rupture 8 cases (1,52%). Post-partum complications were hemorrhage 60 cases (11,4%) and urogenital infections 96 cases (18,25%).

**Discussion:** Discussion: The low socio-economical status are the main factors associated with teenage pregnancy. The precarious education and lack of family support or access to medical healthcare increase the risks and complications. Educational programs and proper use of contraception can reduce teenage pregnancy rate, while adequate antenatal care and delivery can improve the obstetric and perinatal outcome in teenage pregnancies.

## A63: The decline in cost-effectiveness of drugs sought for reimbursement under the Irish health system over a 9-year period

### Ishtar Redman^1^, Patrick Dicker^2^

#### ^1^Medicine, RCSI, Dublin, Ireland; ^2^Public Health and Epidemiology, RCSI, Dublin, Ireland

##### **Correspondence:** Ishtar Redman

**Introduction:** The National Centre for Pharmacoeconomics (NCPE) evaluates the cost-effectiveness of medicines in Ireland following applications for reimbursement by pharmaceutical companies under the Community Drugs Scheme. Following a Rapid Review, drugs with significant budget impact or where value for money is a concern, are further subject to a formal pharmacoeconomic assessment (Health Technology Assessment- HTA). Outcomes of rapid reviews and HTA reports are available to the public (www.ncpe.ie). The objective of this study was to report on the trends in cost-effectiveness as provided in submissions over the period 2009-2017.

**Methods:** A database on all submissions from 2009-2017 was created, including ICER/QALY values extracted from individual HTA reports, as provided by companies seeking reimbursement. Submissions were classified according to therapeutic area and descriptive statistics are presented.

**Results:** A total of 355 dossiers were submitted for the period 2009-2017 and of these, 148 (42%) were subject to full pharmacoeconomic evaluation. The annual number of submissions subject to review steadily increased from 8 in 2009 to 65 in 2017. Associated with this, was a significant expansion in the number of therapeutic areas targeted (from 6 to 15) in addition to a greater diversity in drug-targets within therapeutic areas. The percentage of submissions exceeding the HSE threshold of €45,000/QALY increased from 18% in 2009/2010 to 86% in 2017. Even if the threshold was doubled to €90,000/QALY, 71% exceeded this threshold in 2017.

**Discussion:** In this novel analysis of NCPE data, the cost-effectiveness of drugs sought for reimbursement by pharmaceutical companies has declined sharply over the time period investigated. These results suggest that new models of reimbursement are highly desirable for the Irish health system.

## A64: Medication safety in neonates

### Justin Blaha^1^, Fiona O'Brien^1^, Kamelia Krysiak^1^, Naomi McCallion^2^, Brian Cleary^1,2^

#### ^1^Department of Pharmacy, The Royal College of Surgeons, Dublin, Ireland; ^2^Department of Pharmacy, Rotunda Hospital, Pharmacy, Dublin, Ireland

##### **Correspondence:** Justin Blaha

**Introduction:** Neonatal patients frequently receive continuous administration of drugs through central venous catheters in the neonatal intensive care unit (NICU) (1). This study uses literature review to examine parameters of IV infusion architecture as well as visual and laboratory methods to test the validation of methylene blue (MB) as a model drug.

**Methods:** The validation of MB was accomplished through observation of drug delivery via 4F double lumen venous catheter. Three syringes were placed into B Braun pumps, to model practice at Rotunda Hospital. Syringes containing 50 μg/ml and 10 μg/ml of MB in deionised (DI) water were infused at 0.5 ml/hr. In addition 5% Glucose was infused at 1 ml/hr to represent carrier fluid. A UV detection method for MB was validated in the laboratory. Average absorption values were taken (n=3) over three days for MB at concentrations between 0.15 - 7.5 μg/ml. These concentrations were used to calculate stability over time.

**Results:** The infusion test showed visible accumulation in the octopus and stopcock regions. The flow rate 2 ml/hr showed slow response to the collection point. Laboratory methods revealed MB stabile as a model drug. MB in DI water showed linear absorption: R2=0.99, LOD=0.295, LOQ=0.894. MB in 5% glucose showed linear absorption: R2=0.99, LOD=0.242, LOQ=0.733. MB was shown to retain absorption values over a 72 hr period in both DI water and Glucose.

**Discussion:** The validation of MB as a model drug gives both a visual and stable solution for further tests on flow rate and line architecture.

## A65: Role of P53 in prediction of progression of superficial bladder cancer

### Aladin Mrzic^1^, Senad Bajramovic^2^, Zahid Lepara^2^, Hajrudin Spahovic^2^, Melina Mackic^3^

#### ^1^Medicine, Sarajevo School of Science and Technology, Sarajevo, Bosnia and Herzegovina; ^2^Clinical Center university of Sarajevo, Urology clinic, Sarajevo, Bosnia and Herzegovina; ^3^Sarajevo School of science and Technology, Medicine, Sarajevo, Bosnia and Herzegovina

##### **Correspondence:** Aladin Mrzic

**Introduction:** Evaluation of p53 overexpression can helps us to determine early progression and recidivism of superficial bladder cancer.

**Methods:** We have done open prospective study of 243 patients both male and female age ranged from 49-78 years with diagnosed transitional cell carcinoma (TCC) of bladder after initial TURBT. Additionally p53 overexpression was assessed by immunohistochemistry (IHC). We have focused our study to pT1 grade 2 TCC of bladder and correlation to p53 overexpression. Patients were divided into two groups with positive p53. Group (A)-less than 20% of tumor cells positive to p53 and group (B)-more than 20% tumor cells positive to p53. Patients with multilocular tumor and tumor greater than 3 cm were excluded. Follow up was regularly done by urine cytology and cystoscopy after 3 months regularly for the first year. For comparisons and correlation of the parameters we used chi-square test, Pearson’s index, Fischer’s and Wilcoxon test and multivariate analysis (p<0,05).

**Results:** Thirty six patients were diagnosed with pT1 grade 2, all less than 3 cm and unilocular tumor and positive p53. In group A we had 20 patients who had recidivism of the disease after 9 months into the same grade. In group B we had 16 patients who had recidivism after first 3 months follow up and eight of them had progression into pT2 TCC of bladder (p<0,05).

**Discussion:** Immunohistochemistry approved presence of p53 of tumor cell of TCC of bladder is in corelation with early and rapid recidivism and progression of disease.

## A66: Formulation and assessment of a radiopaque hyaluronic acid hydrogel for in situ imaging

### Hazel Walshe^1^, Helena Kelly^2^, Liam McDonough^2^, Dr. Scott Robinson^3^, Prof. Garry Duffy^3^

#### ^1^School of Pharmacy, Royal College of Surgeons in Ireland, Cork, Ireland; ^2^Royal College of Surgeons in Ireland, School of Pharmacy, Dublin, Ireland; ^3^National University of Ireland in Galway, School of Medicine, Galway, Ireland

##### **Correspondence:** Hazel Walshe

**Introduction:** A bioartificial pancreas is a novel therapeutic approach to the treatment of diabetes mellitus. It seeks to deliver pancreatic islets or glucose responsive stem cells to a protective device, eliminating the need for immune suppression required under current islet transplantation protocols. These islets are often encapsulated in a biocompatible hydrogel for delivery, which must be of sufficient viscosity to suspend islets but also be shear thinning to enable injection via a catheter to the device in situ.

**Methods:** Hyaluronic acid (HA) is the base hydrogel material being evaluated in our system for islet/cell encapsulation. To evaluate its injectability and filling into a proprietary device, a radiopaque hydrogel was formulated to enable imaging of the process while maintaining the desired physical characteristics of the original hydrogel. Visipaque™ 320 was incorporated at a range of concentrations and flow properties, viscosity and gel structure before and after the addition of Visipaque™ 320 were characterized using an AR2000 Rheometer. Once a comparable formulation was identified, the HA-Visipaque™ hydrogel combination was delivered via catheter to the device and this process was imaged in situ using CT in a pig model.

**Results:** All formulations displayed shear thinning behaviour, however the incorporation of Visipaque™ 320 caused a reduction in HA hydrogel viscosity. This required an increase in HA concentration to achieve comparable material characteristics to the original hydrogel used for islet encapsulation.

**Discussion:** This Visipaque™ HA hydrogel combination was delivered by injection via catheter to the device in situ with CT imaging indicating the gel displayed the desired flow properties.

## A67: Torque teno virus load as biomarker for development of BK viremia in renal transplant recipients

### Aline van Rijn^1^, Mariet Feltkamp^2^, Herman Wunderink^3^, Caroline de Brouwer^2^, Joris Rotmans^2^

#### ^1^Medical Microbiology, Leiden University Medical Center, Leiden, Netherlands; ^2^Leiden University Medical Center, Medical Microbiology, Leiden, Netherlands; ^3^University Medical Center Utrecht, Medical Microbiology, Utrecht, Netherlands

##### **Correspondence:** Aline van Rijn

**Introduction:** Torque teno virus (TTV) is a highly prevalent, non-pathogenic human virus. TTV load has been proposed as a biomarker of immunity, for instance in long-term immunosuppressed kidney transplantation (KTx) patients. In KTx patients, over-immunosuppression promotes infection, such as BK polyomavirus (BKPyV)-associated nephropathy which endangers allograft survival. The aim of this study was to explore whether TTV loads can serve as predictor of BKPyV viremia in KTx recipients.

**Methods:** Kidney rejection and BKPyV viremia were retrospectively assessed in cohort of 389 KTx recipients, with one year follow up. BKPyV as well as TTV viral load was determined using real-time PCR in plasma and serum samples taken at five timepoints. Analysis with a joint model for repeated measures and longitudinal data was performed in R.

**Results:** During follow-up, 105 (27%) recipients developed BKPyV viremia. TTV detection increased from 84% at baseline to 100%, which peaked at Month 3 (log 6.9 copies/ml, SD 2.2). KTx recipients who tested TTV positive prior to KTx had a HR of 2.0 (CI: 1.0-4.0) for developing BKPyV viremia. Preliminary analysis showed a modest association between TTV load and development of BKPyV viremia.

**Discussion:** TTV positivity and load correspond to risk of infection in KTx recipients. Further evaluation of TTV as biomarker in KTx recipients seems useful for improving individual treatment strategies.

## A68: Thermal degradation of artificial Sweeteners in baked products

### Jasmine Proudian

#### Medicine, Royal College of Surgeons in Ireland, Dublin, Ireland

**Introduction:** Artificial sweeteners, such as aspartame, sucralose, and saccharin, have no effect on blood sugar levels, making them a viable sugar substitute for diabetics; however, they are susceptible to thermal degradation. The aim of this research study is to determine if artificial sweeteners maintain their sweetness succeeding heat exposure when baked.

**Methods:** 10 males and 10 females between the ages of 15 to 17 were given four unlabelled dishes and cake samples. Each cake was baked with either sucrose (Lantic Sugar), aspartame (Equal), sucralose (Splenda), or saccharin (Sweet’N Low), and each dish contained the granulated form of one sweetener (variables). Sucrose was the control sample and was used as a baseline for comparison. Participants tasted each granulated and cake sample, following each with a water biscuit for palate cleansing (control). To collect statistics, participants rated each sample on a scale from 0 to 3, 0 being “not sweet” and 3 being “very sweet.”

**Results:** The mean sweetnesses of sucrose, aspartame, sucralose, and saccharin in granulated form were 1.45, 2.0, 2.05, and 2.2, respectively, while in baked samples they were 1.35, 2.25, 0.4, 2.15, respectively. Sucrose decreased 6.89%, saccharin decreased 2.27%, sucralose decreased 80.49, and aspartame increased 12.5% in sweetness when baked.

**Discussion:** Aspartame and saccharin would be suitable sugar substitutes in baked goods for diabetics. The preeminent limitation that may have influenced the data was that a taste panel was not used; taste panels undergo training to ensure unbiased information is provided.

## A69: To study the prevalence of depression in pregnant women with gestational diabetes mellitus and its association with metabolic profile

### Aditya Rana^1^, Amitabh Saha^2^

#### ^1^Graduate Wing, Armed Forces Medical College, Pune, India; ^2^Armed Forces Medical College, Psychiatry, Pune, India

##### **Correspondence:** Aditya Rana

**Introduction:** Previous literature suggests, there exists a bi-directional relationship between diabetes and depression.But evidence about prevalence of depression in GDM patients in India and its association with the sugar laboratory profile and anthropometrics is scanty. Objectives of the study were to find the prevalence and association of depression during pregnancy with GDM, metabolic parameters like BMI, Glucose tolerance and BP.

**Methods:** In this cross-sectional analytical study, done at the obstetrics OPD of a hospital, on 347 antenatal cases(gestational age > 24 weeks), socio- demographic details, BP,GDM,BMI,Blood Sugar(OGTT) were measured. For depression status’s assessment, CUDOS(Clinically Useful Depression Outcome Scale)Questionnaire used. Data analyzed by SPSS 23.

**Results:** Significant difference found in the prevalence of depression in GDM patients(56.07%),as compared to Non-GDM(38.5%).And GDM subgroup had more women with mild and moderate depression. Also, Depression and GDM were significantly associated.GDM group had higher mean age and BMI.BMI significantly associated with depression in GDM.GDM aged > 30 were more prone to develop depression. Mean OGTT values were higher among depressed (significant difference for 1hr and 2hr OGTT sample). Also, significant association between the deranged OGTT values above the diagnostic cutoff of GDM and the depression. Middle and high socio-economic group chiefly constituted GDM sub- group.

**Discussion:** Early screening for depression in GDM patients must be done, to manage diabetes well and prevent the development of complications

## A70: Differences between how pregnant women and family members make trade-offs between maternal and fetal health during patient-preference studies

### Oluwabunmi Adesanya^1,2^, Katarina Anderejevic^1,3,4^, Danielle Wuebbolt^1,2^, Rohan D’Souza^1^

#### ^1^ Division of Maternal and Fetal Medicine, Department of Obstetrics & Gynaecology, Mount Sinai Hospital, University of Toronto, Canada; ^2^ Royal College of Surgeons in Ireland, Dublin, Ireland; ^3^ Western School of Medicine, London, Canada; ^4^ University of Toronto, Toronto, Canada

##### **Correspondence:** Oluwabunmi Adesanya

**Introduction:** Our objective was to observe how pregnant women and family members made trade-offs between maternal and fetal health when interviewed independently and together, in patient-preference studies involving the mother-fetus dyad.

**Methods:** A cross sectional study on pregnant women and their family members was conducted at Mount Sinai Hospital, Toronto, Canada. Participants were presented individually, then in pairs, with 7 scenarios comprising a combination of maternal (perfect health or blood clot) and fetal (perfect health, minor or major congenital malformation or death) health states. Outcome preferences were evaluated using a rating scale and the standard gamble method. Comments made during all interviews were recorded and thematically analyzed.

**Results:** 32 pregnant women and 32 family members completed the interviews. In general, preference values obtained through shared interviews were closer to the family member’s than to those of the pregnant women. Disagreement between pregnant women and family members was highest (59%) for the state of ‘perfect maternal health and fetal death’ and least (29%) for ‘maternal blood clot and fetal death’. Common themes included guilt and helplessness around making trade-offs, compromised maternal parenting ability if affected by a clot and the child’s quality of life vs. fetal death. Preference values assigned often did not reflect the comments made.

**Discussion:** Pregnant women and family members find it challenging to make medical decisions involving trade-offs between maternal and fetal health. Shared preference values are closer to those of family members than pregnant women. There is discordance between preference values assigned and observed behavior while eliciting preferences.

## A71: Demographic and fertility outcomes of men seeking fertility assessment

### Amy Pawson^1^, Sergey Moskovtsev^2,3^, Trevor Partch^2^, Sophia Zheng^2^, Karen Lockyear^2^, Pamela Kurjanowicz^,2^, Sarah Kimmins^,4^, Clifford Librach^,3^

#### ^1^Royal College of Surgeons in Ireland, Dublin, Ireland ; ^2^CReATe Fertility Centre, Andrology Department, Toronto, Canada; ^3^Department of Obstetrics and Gynecology, University of Toronto, Canada ; ^4^Department of Pharmacology and Therapeutics, Faculty of Medicine, McGill University, Montreal, Canada

##### **Correspondence:** Amy Pawson

**Introduction:** Little is known about men’s health and its impact on future children. Our long-term goal is to determine mechanisms underlying sperm epigenetic inheritance and disease transmission. This will allow for the development of guidelines for pre-conception nutrition and lifestyle.

**Methods:** Patients were recruited between February 2016-June 2017. Data regarding lifestyle, diet, reproductive history, overall health, semen and blood was collected. Chart review was performed following recruitment and in one-years time, to assess utilization of fertility services and outcomes.

**Results:** In total 134 males from 26 to 58 years (38.2 ± 5.5) were recruited. 80% had primary infertility. The average BMI was 27.4 kg/m2, 63% were overweight or obese. The rate of alcohol consumption, drug use and smoking was 19%. Most men had a BcS degree (46%) or postgraduate education (44%). Health history was unremarkable in 44%; while 56% had a history of STIs, gonadal injuries, sexual dysfunction, or cofounding medical conditions. 69% had normozoospermia, the remaining at least one abnormal semen parameter. High sperm DNA damage was present in 16% of patients. Since initial consultation, 16% achieved a pregnancy naturally. Intra-uterine inseminations were performed in 28% with cumulative pregnancy rates of 35%. IVF/ICSI was performed in 42% with cumulative pregnancy rate 58%. In addition, 10% are still trying naturally; 3% no longer trying; 10% did not proceed.

**Discussion:** Males presenting for treatment were well-educated, overweight, suffering from higher rates of medical conditions and suboptimal semen parameters. 52% of couples achieved a pregnancy in the one-year period after recruitment. IVF/ICSI had the highest success.

